# The specificity and structure of DNA crosslinking by the gut bacterial genotoxin colibactin

**DOI:** 10.1101/2025.05.26.655968

**Published:** 2025-05-27

**Authors:** Erik S. Carlson, Raphael Haslecker, Chiara Lecchi, Miguel Aguilar Ramos, Vyshnavi Vennelakanti, Linda Honaker, Alessia Stornetta, Estela S. Millán, Bruce A. Johnson, Heather J. Kulik, Silvia Balbo, Peter W. Villalta, Victoria D’Souza, Emily P. Balskus

**Affiliations:** 1 Department of Chemistry and Chemical Biology, Harvard University, Cambridge, MA 02138, USA; 2 Department of Molecular and Cellular Biology, Harvard University, Cambridge, MA 02138, USA; 3 Masonic Cancer Center University of Minnesota, Minneapolis, MN 55455, USA; 4 Department of Chemistry, Massachusetts Institute of Technology, Cambridge, MA 02139, USA; 5 Department of Chemical Engineering, Massachusetts Institute of Technology, Cambridge, MA 02139, USA; 6 Structural Biology Initiative, City University of New York (CUNY) Advanced Science Research Center, New York, NY 10031, USA; 7 Department of Medicinal Chemistry, University of Minnesota, Minneapolis, MN 55455, USA; 8 Howard Hughes Medical Institute, Harvard University, Cambridge, MA 02138, USA

## Abstract

Accumulating evidence has connected the chemically unstable, DNA-damaging gut bacterial natural product colibactin to colorectal cancer, including the identification of mutational signatures that are thought to arise from colibactin-DNA interstrand crosslinks (ICLs). However, we currently lack direct information regarding the structure of this lesion. Here, we combine mass spectrometry and nuclear magnetic resonance spectroscopy to elucidate the specificity and structure of the colibactin-DNA ICL. We find that colibactin alkylates within the minor groove of AT-rich DNA, explaining the origins of mutational signatures. Unexpectedly, we discover that the chemically unstable central motif of colibactin mediates the sequence specificity of crosslinking. By directly elucidating colibactin’s interactions with DNA, this work enhances our understanding of the structure and genotoxic mechanisms of this unique cancer-linked gut bacterial natural product.

## Introduction

The human gut microbiome has been increasingly linked to the development of colorectal cancer (CRC) (1,2). Particularly prominent potential contributors to this disease are gut bacteria that produce colibactin (3.^4^). Colibactin is a complex, chemically unstable genotoxic natural product produced by commensal Enterobacteriaceae, including strains of *E. coli,* that harbor the *pks* (or *clb*) gene cluster (5). This gene cluster encodes a biosynthetic pathway that employs a nonribosomal peptide synthetase-polyketide synthase assembly line. Exposure of human cells to *pks*^+^ bacteria results in DNA damage, including double strand breaks (DSBs) (5). This gives rise to various phenotypes *in vitro* and *in vivo* including genomic instability, megalocytosis, G2/M cell cycle arrest, cellular senescence, and increased tumor formation in mouse models of CRC (5-11). Importantly, *pks*^+^ *E. coli* are detected more frequently in CRC patients (8,9,11-14), fueling the hypothesis that colibactin exposure may play a role in the initiation and/or progression of cancer.

**Fig. 1.**
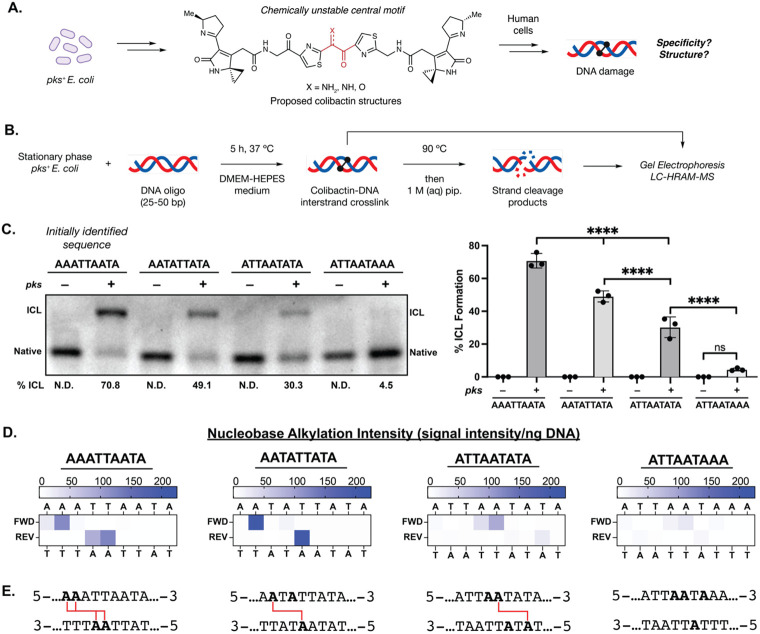
The chemically unstable gut bacterial genotoxin colibactin forms DNA interstrand crosslinks (ICLs) in a sequence specific manner. (A) The chemical structure of colibactin and the structure and specificity of the colibactin-DNA ICL are unresolved. (B) Experimental workflow for characterizing ICL formation on 25-50 bp double-stranded oligonucleotides using colibactin produced in situ by *pks*^+^ *E. coli.* (C) Extent of ICL formation within 50mers possessing the indicated sequence motifs. Crosslink formation was identified by denaturing gel electrophoresis and quantified by densitometry. Quantified values are displayed as a column plot. (D) Residue-specific alkylation of 25mers containing the indicated sequence motifs. Alkylation intensities were determined through liquid chromatography-high resolution accurate mass-mass spectrometry (LC-HRAM-MS) analysis and normalized to total DNA injected. Intensity reported is the difference between the average detected in assays with *pks*^+^ *E. coli* compared to the average detected in assays with *pks^−^ E. coli.* (E) Inferred ICL locations within tested sequence motifs. All alkylated residues are bolded and those crosslinked are connected by red lines. All data are mean +/− s.d. with n=3 biological replicates. Full 50 bp and 25 bp sequences are available in SI. N.D.: Not detected.

Understanding the molecular basis of colibactin’s genotoxic activity has been particularly challenging because this natural product has been recalcitrant to traditional isolation and structure elucidation approaches. Studying biosynthetic enzymes and identifying shunt products from *pks* mutant strains revealed structural information, including the unexpected incorporation of cyclopropane rings into colibactin, leading to the proposal that it directly alkylates DNA (15-17). Subsequent discovery of colibactin-derived DNA adducts and the observation that *pks*^+^ *E. coli* generate DNA interstrand crosslinks (ICLs) *in vitro* and in cell lines further supported this hypothesis (18-20). Additional biochemical studies, chemical synthesis, and isolation attempts ultimately led to the proposal that colibactin is a pseudosymmetric molecule containing two electrophilic cyclopropane ‘warheads’ capable of alkylating DNA at adenine (Ade) (Fig. 1A) (21,22). These warheads are connected by a central scaffold of unresolved structure that is predicted biosynthetically to be an α-aminoketone (or its enolamine tautomer); however, this motif likely undergoes rapid oxidation to the corresponding α-ketoimine, followed by hydrolysis to a 1,2-diketone that is susceptible to oxidative C─C bond cleavage (Fig. S1A) (23). This highly unstable central structural motif has been replaced by two methylene groups in a ‘stable’ synthetic colibactin analog (Fig. S1B) (24). However, this analog is a minor component of an inseparable product mixture, with the major components being two β-hydroxy lactam ring diastereomers of unknown biological significance. Though the proposed structure(s) of colibactin account for the activities of all essential biosynthetic enzymes and explain ICL formation, important gaps in our understanding of its structure and activity remain, including the identity and function of its central scaffold.

The molecular details underlying colibactin’s interaction with DNA are also unclear. The discovery of mutational signatures arising from exposure of human cells and organoids to *pks*^+^ *E. coli* has provided intriguing indirect insights (25,26). These mutational signatures are primarily T>C single-base substitutions and indels that occur within AT-rich sequence motifs (i.e. AAWWTT), and their transcriptional-strand bias is consistent with ICL formation between two adenines. Colibactin-induced DSBs occur within identical AT-rich sequences and are suggested to arise from degradation of ICLs (26). Additionally, molecular modeling suggests the colibactin-DNA ICL spans 4-5.5 Å (or 3-4 bp) but could not elucidate the specific adenines alkylated or the molecular details of DNA binding (26). Colibactin mutational signatures have been detected in many cancer genomes, including 5-20% of CRC genomes, occur in driver genes such as *APC,* and are correlated with early-onset CRC (25-33). We also recently identified colibactin-DNA adducts in human colonoscopy samples (34). Together, these data indicate that humans are exposed to colibactin, strengthening its connection to cancer development. They also provide indirect evidence that colibactin crosslinks DNA in a highly specific manner. However, we currently lack direct information regarding the specificity and structure of the colibactin-DNA ICL, limiting our understanding of how this natural product targets DNA and the origins of mutational signatures arising from this DNA damage.

Here, we experimentally elucidate the specificity and structure of the colibactin-DNA ICL. By investigating colibactin’s reactivity toward oligonucleotides, we show that it binds in the minor groove of DNA and forms bis-*N*3-dAdo ICLs within a preferred motif of 5′-W**A**WWTW-3′ (where the adenines bolded and opposite the underlined thymine are alkylated) that is consistent with previously reported mutational signatures. We also solve the first solution-state structure of a colibactin-DNA ICL using high-resolution NMR spectroscopy, providing direct information about colibactin’s structure and the basis for ICL formation. Unexpectedly, mass spectrometry and NMR reveal an α-ketoiminium in the central region of colibactin that likely serves as a key DNA recognition element, explaining colibactin’s sequence selectivity. Together, these biochemical and structural data reveal a strategy for DNA alkylation unique among natural products, greatly enhancing our understanding of colibactin’s chemical structure, its recognition of and reaction with DNA, and its downstream effects on the host genome.

## Results

To study colibactin-DNA ICL formation in detail, we initially identified a short, double-stranded oligodeoxynucleotide (dsODN) that was crosslinked upon exposure to *pks*^+^ *E. coli.* We generated colibactin *in situ* from bacterial metabolism because of the prominent gaps in our understanding of this metabolite and the concern that synthetic analogs may differ in activity and/or selectivity. Examining colibactin’s reactivity toward plasmid fragments identified a 500 bp region of pET28(a) that was crosslinked efficiently. Systematically truncating this 500mer identified a 50mer that is crosslinked (71% ICL formation) after a 5 h incubation at 37 °C with *pks*^+^ *E. coli* in DMEM-HEPES medium, pH 7.4 (Fig. 1B-C). No ICL formation was observed when incubating the 50mer with an isogenic *pks^−^ E. coli* strain. We noted that this 50mer contained an AT-rich motif (5′-AAATTAATA-3′) that is consistent with previously reported colibactin mutational signatures (25,26), leading us to hypothesize colibactin directly forms ICLs within this region.

To determine the site(s) of alkylation within this sequence motif, we developed a strand cleavage assay utilizing liquid chromatography-high-resolution accurate mass-mass spectrometry (LC-HRAM-MS) for detection (Fig. 1B, Fig. S2). Colibactin-ICLs are thermally unstable and spontaneously depurinate to form abasic sites when heated to 90 °C (35,36). Subsequent base treatment induces strand cleavage at the colibactin-specific abasic sites, and the masses of the resulting strand cleavage products are determined by isotopically-resolved mass deconvolution of the LC-HRAM-MS data to identify the location and measure the relative abundance of DNA alkylation (Fig. 1D-E). We applied this assay to a *pks*^+^ *E. coli*-exposed 25mer containing the AT-rich motif identified from the 50mer. This shortened dsODN was chosen to increase the analytical capability of the LC-MS analysis. The results indicate site-specific alkylation of this dsODN by colibactin at 5**′-AA**ATTAATA-3′ (where alkylated nucleotides are bolded or opposite to underlined nucleotides) (Fig. 1D-E, Fig. S2,3). The accuracy of our LC-MS approach was confirmed by subjecting a 5′-6-carboxyfluorescein-labeled 50mer to our crosslinking and strand cleavage protocol and determining alkylation site locations using traditional Maxam–Gilbert gel sequencing (Fig. S4-6) (37).

This result suggests the colibactin-DNA ICL spans 3-5 bp. To determine the precise length of the ICL, we repeated our crosslinking and strand cleavage assays with a 50mer and 25mer containing the sequence 5′-AATATTATA-3′ which has the potential to form ICLs between pairs of adenines 2 – 8 bp apart. The results of this assay indicated colibactin forms one unique ICL between two adenines spanning 4 bp (5′-A**A**TATTATA-3′). This is consistent with previous studies showing colibactin-induced DSBs have 2 bp overhangs (26) and suggests that the four sites of alkylation within the 5′-AAATTAATA-3′ motif we initially tested likely correspond to a mixture of two colibactin-DNA ICLs (Fig. 1D-E, Fig. S4). The extent of ICL formation was reduced compared to the initial sequence perhaps due to this sequence possessing only one recognition site (Fig. 1C-E).

We further explored the position of colibactin-DNA ICL formation by performing incubations with dsODNs containing A to T transversions at the previously observed sites of alkylation (5′-ATTAATATA-3′). If colibactin can alkylate at the same position but on the opposite strand, we would expect ICL formation in the same location (i.e. 5′-ATTA**A**TATA-3′); if not, we would expect the ICL to shift to a new location (5′-ATTA**A**TATA-3′). ICL formation and strand cleavage analysis revealed the ICL in the new location, suggesting colibactin preferentially crosslinks at a TA base pair three positions downstream from the initial A (Fig. 1C-E). Finally, we detected little to no alkylation of dsODNs containing 5′-ATTAATAAA-3′, which has no 5′-AWWT-3′ motifs (Fig. 1C-E). Taken together, this data suggests that colibactin-DNA ICLs form at 5′-W**A**WWTW-3′ motifs.

We next introduced single GC base pairs into the 5′-AATATT-3′ motif to test colibactin’s specificity for adenine alkylation (Fig. S7A) and tolerance for changes to the surrounding sequence. It has been postulated that colibactin may not recognize GC-containing sequences due to their altered DNA groove widths (26). Single GC base pair-containing sequence variants were largely crosslinked at comparable levels to the parent sequence except for 5′-AGTATT-3′ and 5′-AATACT-3′, which contain a substitution at an alkylation site, and 5′-AATATC-3′ which contains a substitution at an outer base pair flanking this site (Fig. S7B). Strand cleavage analysis revealed sites of alkylation identical to those of the parent sequence, except for 5′-AGTATT-3′ and 5′-AATACT-3′. Though no alkylation was observed at the newly incorporated guanine in these sequences, formation of colibactin-DNA monoadducts was observed at the adenine on the other strand (Fig. S7C-D). Altogether, these studies directly support colibactin forming bis-Ade ICLs within sequence motifs matching previously reported sites of DSBs and mutational signatures, strengthening the evidence that colibactin ICLs are the inciting incidents to these downstream events. Our results also reveal that colibactin can react within a broader sequence context than suggested by the mutational signatures.

**Fig. 2.**
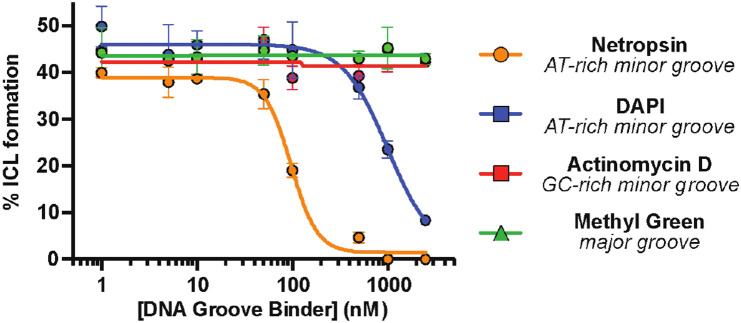
Colibactin binds AT-rich regions of the minor groove. ICL formation by *pks*^+^ *E. coli* in the presence of select DNA groove binding small molecules. Dose-dependent inhibition of ICL formation by AT-rich minor groove binders was observed by denaturing gel electrophoresis and quantified by densitometry. Data are mean +/− s.d. with n = 3 biological replicates.

With direct evidence that colibactin alkylates AT-rich DNA in a sequence-specific manner, we next sought to elucidate its groove specificity. Based on the structural characterization of colibactin-DNA adducts, colibactin is thought to alkylate *N3* of adenine, which is accessible via the minor groove of DNA (18). Prior calculations also suggested that shape complementarity and electrostatics could lead to a preference for minor groove binding (26). We directly tested this proposal by incubating the 50mer containing the 5′-AAATTAATA-3′ motif with *pks*^+^ *E. coli* in the presence of DNA-binding small molecules possessing differing topological preferences (Fig. S8) after confirming these molecules had no effect on bacterial growth or colibactin production (Fig. S9-10). AT-rich minor groove binders (netropsin and DAPI) (38,39) inhibited ICL formation in a dose-dependent manner (Fig. 2). By contrast, a major groove binder (methyl green) (40) and a GC-rich minor groove binder (actinomycin D) (41) showed no effect. These results provide strong experimental evidence that colibactin specifically alkylates AT-rich minor grooves.

We next investigated the site of colibactin DNA alkylation on adenine. As highlighted above, we previously characterized an *N*3-Ade colibactin-DNA adduct (18). Additional Ade adducts containing the other ‘half’ of colibactin were identified using mass spectrometry but were not characterized by NMR spectroscopy, leaving their connectivity unknown (21,22). Hypothesizing that both colibactin cyclopropanes alkylate the *N*3 position of adenine, which would be consistent with selective minor groove binding, we incubated *pks*^+^ *E. coli* with 50mers containing a 5′-A**A**TATT-3′ sequence motif in which the bolded residue was replaced with *N*3-deaza-dAdo (42), eliminating the possibility for *N*3-dAdo adducts to form. ICL formation was abolished when *N*3-deaza-dAdo was incorporated into the forward, reverse, or both strands (Fig. S11). Strand cleavage analysis of assays with 25mers containing these sequence variants also showed no alkylation of *N*3-deaza-dAdo, instead revealing monoadduct formation for the singly substituted variants. The presence of monoadducts was confirmed using the LC-HRAM-MS cleavage assay (Fig. S11C-D). Taken together, these data indicate colibactin exclusively forms bis-*N*3-dAdo ICLs within the minor groove of AT-rich DNA.

**Fig. 3.**
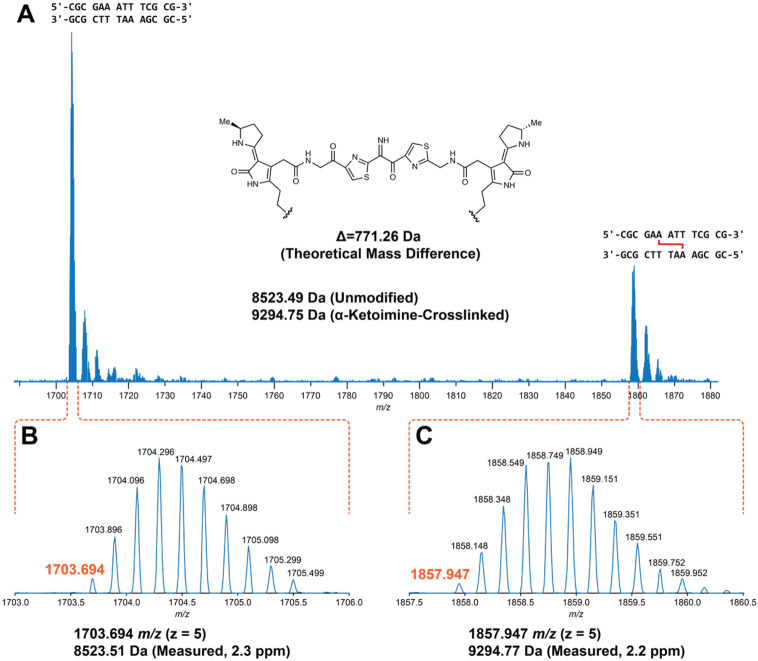
Mass spectrometry analysis of an intact ICL reveals a central α-ketoimine in colibactin. HRAM LC analysis of crosslinked 14-mer DNA double strand oligonucleotide exposed to *pks*^+^ *E. coli.* (A) Full scan spectrum containing −5 charge state signal of unmodified and modified 5’-CGCGAAATTTCGCG-3’ double strand oligonucleotides. (B) Expanded region of spectrum corresponding to the unmodified oligonucleotide. (C) Expanded region of the spectrum corresponding to the modified oligonucleotide.

To gain initial insights into the structure of the colibactin-DNA ICL, we characterized 14 bp and 25 bp oligo substrates containing previously examined sequence motifs with LC-HRAM-MS after *pks*^+^ *E. coli* incubation (Fig. 3). For each dsODN that became crosslinked, we observed a mixture of native and modified dsODNs. After direct deconvolution analysis of the 14 bp dsODN samples, we measured a mass difference of *m/z* 771.26 (Fig. 3). The 25 bp dsODN samples required additional analysis and provided mass differences consistent with the value measured for the 14 bp samples (Fig. S12, Supplementary Discussion). We did not observe any other species in these assays. All dsODNs incubated with *pks^−^ E. coli* showed no mass shift. The observed mass is consistent with formation of a single ICL arising from the proposed colibactin structure containing an α-ketoimine. This unexpected observation sharply contrasts with the structures proposed for colibactin detected in aqueous solution and in putative colibactin and ICL degradation products, which all contain a 1,2-diketone (21,22,43). Additionally, the observed mass of colibactin indicates no oxidation of the ring-opened electrophilic warheads, unlike in previously characterized colibactin-DNA adducts (18-21). This direct characterization of the colibactin-DNA ICL via MS helps to resolve important aspects of colibactin’s structure.

To understand the molecular details of how colibactin binds and crosslinks DNA, we used solution-state NMR. This required generating the colibactin-DNA ICL on a large scale using *pks*^+^ *E. coli.* We chose a 14mer dsODN with a palindromic sequence (5′-CGCGA**A**TATTCGCG-3′) and substituted A6 with 2′-fluoro-deoxyadenosine to increase the glycolytic bond strength and minimize depurination (44). To access sufficient quantities of the colibactin-DNA ICL (~15 nmol), hundreds of small-scale incubations were performed in 96-well plates, combined, and purified to provide a mixture of crosslinked DNA (~55-65%) and free DNA. Thus, we first assigned the spectra of free DNA (with and without 2′-fluoro-A6 modification), which allowed for comparative, unambiguous assignments of DNA chemical shifts that are perturbed upon crosslinking to colibactin (Table S1). Overall, we do not observe any significant changes in base-to-ribose NOE walks throughout the DNA molecule, indicating that the overall B-form groove parameters are maintained upon colibactin crosslinking. This observation suggests that the shape of colibactin is complementary to that of the minor groove.

The structural data verified the sites of alkylation by colibactin identified in our earlier experiments. In comparison to free DNA, the aromatic ring protons of A6_a_ and the equivalent A6_b_ (complementary strand) residues had the largest chemical shift change, with the H2 protons moving downfield by ~0.36 and ~0.26 ppm, and the H8 by ~0.83 and ~0.78 ppm, respectively (Fig. S13A). In contrast, the average change for the other 2′-deoxyadenosines in the DNA was less than 0.04 ppm. While as expected, the majority of interactions between colibactin and DNA are located at the ATAT sequence where colibactin alkylates, interactions were also observed from the flanking base pairs, A5_a_-T10_b_ and T10_a_-A5b, indicating an expanded recognition motif.

**Fig. 4.**
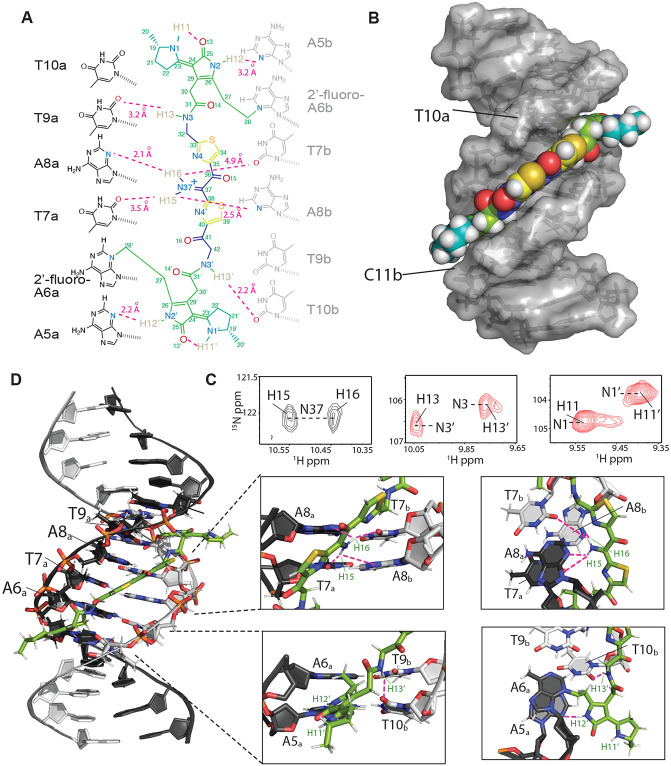
Structure of the colibactin-DNA ICL. (A) Schematic and atom numbering of colibactin, and its hydrogen bonding and electrostatic interaction with nucleotide bases as denoted by magenta dashed lines. (B) Surface representation of the structure showing the pyrrolidine, thiazole and pyrrole rings stacked in between the minor groove edges of the DNA parallel to the long axis. (C) Portions of a 2D HSQC experiment with a [^15^N,^13^C]-colibactin-DNA ICL sample showing two hydrogen atoms correlated with N37 indicating protonation of the nitrogen (left). The equivalent N3/3’ (center) and N1/1’ (right) nitrogens and their associated protons have different chemical shifts indicating different environments and the pseudo-symmetric nature of the interaction. (D) Cartoon representation of the interaction and zoom-in views showing the placement of, i) colibactin iminium moiety with the electronegative atoms of the center T7A8 residues of the DNA (top), and ii) the amide hydrogen (H13) and the pyrrolidinone ring hydrogen (H12) interacting with T10b and A5a nucleobase (bottom). For clarity, only the inner motif residues (A6-T9) on the A-strand have been labeled.

The pseudosymmetric nature of the colibactin-DNA ICL was also readily discernible. First, equivalent protons from both the colibactin and the palindromic DNA have distinct chemical shifts, for example, the equivalent A6a and A6b H8 differ by 0.05 ppm, indicating that they are in slightly different environments (Fig. S13A). Second, while such equivalent protons gave rise to similar NOE connectivities with protons in close proximities, the NOEs have slightly different intensities, indicating the same interactions but at different distances (Fig. S14). Overall, with the exception of the pyrrolidine rings at either end of colibactin, we observe NOEs from almost all of its protons to the extended AATATT sequence. The observations that define the orientation of the various colibactin rings with respect to the DNA groove are: 1) no connectivities from the terminal pyrrolidine rings; 2) the thiazole hydrogens C34H and C39H give connectivities to the outer deoxyribose hydrogens 9a and 10b (H4'/H5'), respectively; 3) the N3H and N3'H amides and hydrogens of C27 and C28 (the carbon attached to the N3 of A6), which flank the pyrrolidinone rings, show connectivities to each other, thus confining the ring inside the groove (Fig. 4B,D, and Fig. S14). Consequently, the structures show that, while the terminal pyrrolidine rings point out of the minor groove, the thiazole rings are wedged in, aligning with the phosphodiester backbone with their bulky sulfur atoms pointing outwards. Likewise, the pyrrolidinone rings of the warheads are stacked parallel to the groove, slightly outside of the AATATT sequence. This results in a tightly packed DNA-colibactin ICL with colibactin in an extended, concave conformation spanning along half a turn of the minor groove (Fig. 4B,D and Fig. S13C). The curved shape and close contacts between its heterocyclic aromatic rings and the hydrophobic surfaces of the minor groove walls suggests shape complementarity as well as van der Waals and hydrophobic interactions all contribute to colibactin-DNA binding.

We also observed hydrogen bonds and/or electrostatic interactions from all nitrogen protons in colibactin which may explain its specificity for the preferred sequence motif. First, while the NMR data confirm the presence of a nitrogen in the central scaffold of colibactin, surprisingly we find this nitrogen within an iminium functional group. Two distinct proton peaks are correlated with N37 in the ^15^N-HSQC experiment, indicating a protonation event at this site (Fig. 4C). Iminium formation may be explained by the central region of the AATATT binding pocket, which comprises the floor of the minor groove and provides a highly electronegative environment for colibactin, with N37 being near O2 of T7 and N3 of A8 of both strands. While each iminium proton gives NOEs to A8H2/H1' protons of both strands, H16 had comparatively stronger NOEs, thus placing the iminium slightly closer to the a-strand of the DNA. The NOE correlations from these protons are thus in line with the iminium forming a hydrogen bond with N3 of A8a and electrostatic interactions with O2 of T7b, O2 of T7, and N3 of A8b. Second, the equivalent colibactin H13 and H13' amide protons show connectivities to the flanking T9a and T10b H1' respectively, suggesting they are positioned to form hydrogen bonding and electrostatic interactions with the O2 position of these thymines. Third, the H12 and H12' protons of colibactin's pyrrolidinone rings, which are the sites of alkylation, also showed connectivities to A5bH1'/C11aH2' and A5aH1'/ T10bH2' indicating that these nitrogens are situated in the center of the groove, electrostatically interacting with the A5 N3 position on either end of the sequence motif (Fig. 4C and S14). This orientation would position the cyclopropane rings of the colibactin warheads in close proximity to N3 of A6. Lastly, although H11 and H11' gave no connectivities to DNA, they were still in slow exchange and gave strong connectivities to the hydrogens in the pyrrolidine rings, indicating that they are most likely forming internal hydrogen bonds to the C25 and C25' carbonyl groups in the adjacent pyrrolidinone rings (Fig. S14). These intramolecular hydrogen bonds may form prior to alkylation, and if so, would place the heterocyclic rings of the electrophilic warheads coplanar, enhancing their electrophilicity. Importantly, we see no evidence for intramolecular cyclization of colibactin (24), indicating the ICL derives from a linear pseudosymmetric structure.

This structural information allows us to formulate a model for colibactin-DNA ICL formation (Fig. S15). We propose that the central region of colibactin, with its concave shape and heterocyclic aromatic rings, facilitates binding within the minor groove of AT-rich DNA sequences. We also predict colibactin-DNA binding will be enhanced by multiple positively charged functional groups. In addition to the positively charged pyrrolinium rings of the two electrophilic warheads, the positively charged nitrogen atom of the central aminoketone, enolamine, or α-ketoiminium of colibactin likely plays a critical role in its selectivity for AT-rich minor groove binding by providing favorable electrostatic interactions and hydrogen bonding to the central base pairs in the 5′-WAWWTW-3′ motif, which form the floor of the minor groove. Finally, the structure also suggests that binding within the minor groove promotes inter- and intramolecular hydrogen bonding and electrostatic interactions involving the colibactin warheads that enhance their electrophilicity and orient the spiro-cyclopropanes and π-systems of the adjacent pyrrolinium heterocycles with the appropriate stereoelectronic alignment to trigger ring opening by N3 of Ade.

**Fig. 5.**
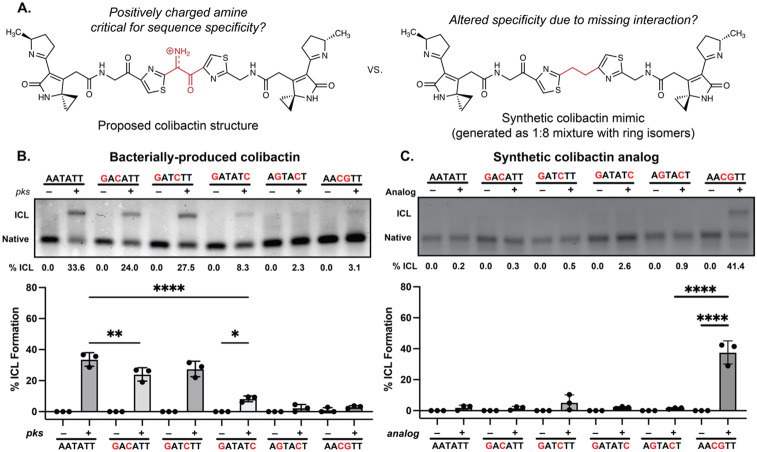
The unstable central motif of colibactin influences its sequence specificity for ICL formation. (A) Structural comparison of bacterially produced colibactin and a synthetic ‘stable’ colibactin analog suggests the analog may have altered specificity for ICL formation. (B) Quantification of ICL formation by co-incubation with bacteria through denaturing gel analysis for 50mers containing a double GC substitution within the sequence 5′-AATATT-3′. Results are quantified through densitometry and shown as bar plots. (C) Quantification of ICL formation by treatment with a colibactin analog through denaturing gel analysis for 50mers containing a double GC substitution within the sequence 5’-AATATT-3’. Results are quantified through densitometry and shown as bar plots. All data are mean +/− s.d. and n = 3 biological replicates. **** P < 0.0001; ** P < 0.01; * P < 0.05; not significant (ns) P > 0.05, one-way ANOVA and Tukey’s multiple comparison test.

To test this model, and specifically the importance of colibactin’s central structural motif to the specificity of ICL formation, we compared its sequence specificity to that of the stable synthetic colibactin analog possessing two central methylene groups (Fig. 5A) (24). We posited that colibactin and the synthetic analog would have different preferences for alkylation of sequences containing multiple CG base pairs within the 5′-WAWWTW-3′ motif. If hydrogen bonding and electrostatic interactions with colibactin’s central nitrogen atom are critical for sequence specificity, exchanging the two central TA base pairs for CG base pairs should diminish alkylation due to the resulting steric hindrance and electrostatic repulsion from the exocyclic amines of the two guanines, which alter the surface of the minor groove floor. By contrast, the stable colibactin analog lacking the central structural motif should tolerate this substitution.

We incubated either *pks*^+^ *E. coli* or the synthetic analog with a series of 50 bp dsODNs that contained two GC base pairs at different positions within a 5′-AATATT-3′ motif and measured ICL formation using gel electrophoresis (Fig. 5B). As predicted, the introduction of two GC base pairs at the center of this motif almost completely abolished alkylation by colibactin. ICL formation was also abolished when GC base pairs were installed at the two sites of alkylation and greatly diminished when GC base pairs were introduced at the two outer positions flanking the alkylation site. Other substitutions had minimal effects on ICL formation. Strikingly, the specificity of the synthetic analog differs from that of natural colibactin (Fig. 5C). We observed a preference for alkylation at the 5′-AACGTT-3′ sequence, which is not targeted by natural colibactin, and minimal alkylation at other sequences. Finally, we compared the reactivity of colibactin and the synthetic analog toward additional oligos containing two GC base pairs in the central positions (5′-AAGCTT-3′, 5′-AACCTT-3′, and 5′-AAGGTT-3′) (Fig. S16). Again, colibactin did not alkylate these motifs while the analog displayed robust ICL formation. These results further support a critical role for the central structural motif and its positively charged nitrogen atom in the selectivity of colibactin ICL formation.

**Figure 6.**
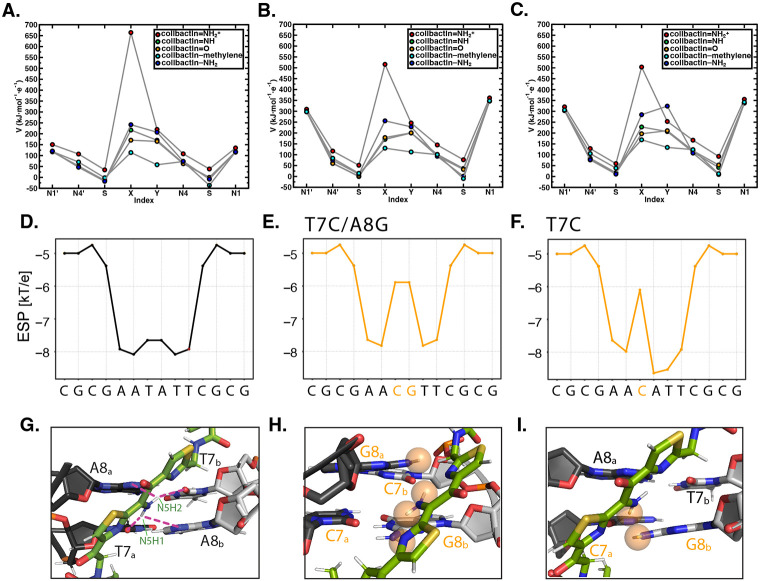
Electrostatic and steric interactions involving colibactin’s unstable central motif likely drive sequence specificity for DNA alkylation. Electrostatic potential (ESP) values of proposed colibactin structures (V in kJ·mol^−1^·e^−1^) obtained from density functional theory (DFT) optimization calculations of proposed colibactin structures at the B3LYP-D3/6-31G* level of theory. ESP values were calculated for (A) proposed structures of free colibactin (B) proposed colibactin structures crosslinked to the doubly charged DNA sequence GAATATTC (C) proposed colibactin structures crosslinked to the doubly charged double mutant DNA sequence 5′-GAACGTTC-3′. Proposed colibactin structures included the α-ketoiminium (colibactin=NH_2_^+^), α-ketoimine (colibactin=NH), diketone (colibactin=O), CH_2_–CH_2_ (colibactin–methylene), and enolamine (colibactin–NH_2_) central functional groups. The indices X and Y correspond to the heavy element indices of the central functional group. X=N and Y=O for colibactin=NH_2_^+^, colibactin=NH, and colibactin–NH_2_. X=O and Y=O for colibactin=O. X=C and Y=C for colibactin–methylene. (D–F) Electrostatic potential calculations using DNAPhi predicting the highly electronegative environment in the sequence containing the preferred AATATT motif (black) and the decrease in electronegativity upon sequence substitution in the central motif (orange). (G-I) Comparison of the NMR structure with structural modeling of the substituted sequences shown in E and F, showing potential steric clashes between colibactin and DNA (orange spheres).

To gain additional insight into the interactions influencing the specificity of colibactin-DNA ICL formation, we calculated the electrostatic potential (ESP) values centered on atoms in proposed colibactin structures bearing different central functional groups, both free and crosslinked to DNA (Fig. 6A and Fig. S17,18). We observe the highest ESP on the iminium nitrogen atom of colibactin bearing a central α-ketoiminium (ca. 664 kJ·mol^−1^·e^−1^), consistent with the α-ketoiminium having a positive charge. We calculate a lower ESP on the iminium nitrogen of the α-ketoiminium when this proposed colibactin is crosslinked to DNA compared to that of the corresponding free colibactin (Fig. 6B-C and Fig. S19,20). This decrease could indicate stabilization by electrostatic or other non-covalent (e.g. hydrogen bonding) interactions with the DNA. However, we do not observe a significant decrease in ESP of the heavy atoms of the central functional groups in the other proposed colibactin structures when they are crosslinked to DNA, suggesting the electrostatic interactions between DNA and colibactin are much stronger for the proposed structure bearing a central α-ketoiminium.

Additional calculations focused on DNA further support the importance of electrostatic interactions to colibactin-DNA recognition. Using DNAPhi to predict the ESP of the minor groove of the dsODN used for our structural studies, we find that, as expected, the center of the 5′-AATATT-3′ motif is substantially more electronegative than the other positions, accounting for the increased pK_a_ of N37 of colibactin and its protonation (Fig. 6D) (45). Substitution of a single central base pair with a CG base pair increases ESP at this position while substituting both central base pairs, which abolished ICL formation by colibactin, results in a loss of this ESP differential (Fig. 6E,F). Modeling interactions between colibactin and these different sequences shows distortions when guanines are introduced due to repulsions from the large amino moieties of these central base pairs (Fig. 6G-I). The outer base pairs flanking the alkylation site (A5_a_ - T10_b_ and T10_a_ - A5_b_) also contribute to the negative ESP, and modeling GC base pair substitutions at these positions also results in distortions and repulsions, potentially disrupting key hydrogen bonding and electrostatic interactions with the warheads and accounting for the reduced ICL formation in these sequences (Fig. S21). In summary, this structure, experimental data, and computations reveal and explain colibactin’s specificity for DNA alkylation, highlighting an especially important role for electrostatic interactions involving its chemically unstable central motif.

## Discussion

Gaining a molecular understanding of DNA-damaging agents is critical for advancing our understanding of their connections to cancer and use as therapeutic agents. As mutational signatures are increasingly identified in cancer genomes (46,47), it is imperative to elucidate their origins. Characterizing the DNA lesions that give rise to specific mutational signatures can provide starting places for investigating repair pathways and processes that lead to misrepair. DNA-targeting small molecules are also an important class of therapeutics (48). Studying the specificities and structures of DNA-binding and -alkylating natural products and synthetic compounds has revealed important chemical principles underlying small molecule-DNA recognition and reactivity (49,50).

Prior to this study, there was little direct structural information about colibactin’s interaction with DNA. Characterization and detection of colibactin-derived DNA adducts had revealed ICL formation likely occurred between two adenines and had revealed N3 of adenine as one site of alkylation (18,19,21,22). The latter observation implied that binding may occur in the minor groove. Discovery of colibactin mutational signatures and the sequence preference of DSB formation later implicated AT-rich sequences as likely sites for colibactin alkylation (25,26). However, because these outcomes occur downstream of ICL formation and in complex cellular settings, they provide only indirect evidence for the colibactin’s sequence specificity. Computational modeling of colibactin’s interactions with DNA further suggested that preferential binding within AT-rich minor grooves might be driven by the properties of these regions of DNA, including their narrow width and negative ESPs (26). However, these calculations could not predict the precise nature of colibactin’s interactions with DNA nor specify the exact sites of alkylation.

By combining MS and NMR methods, we have directly established the specificity and structure of the colibactin-DNA ICL. An LC-MS based assay was created pairing base treatment-induced cleavage of DNA ICL oligonucleotides with mass analysis of the resulting cleavage products to determine the site-specificity of ICL alkylation on a wide variety of sequences. By characterizing colibactin’s reactivity toward dsODNs in vitro, we observed alkylation at a preferred sequence motif of 5′-W**A**WWTW-3′, which corresponds to the locations of reported mutational signatures, illuminating their origin. Intriguingly, we observed a broader capacity for ICL formation than that inferred from prior cell-based experiments, with single GC base pairs largely tolerated in this preferred motif. This could indicate that ICLs in certain sequence contexts are repaired more effectively than others in vivo. Our experiments also reveal the potential for monoadduct formation, which may further contribute to colibactin’s genotoxicity. This information and the identification of conditions for generating well-defined colibactin-DNA ICLs and monoadducts in vitro will enable future efforts to elucidate the specific DNA repair pathways and enzymes involved in responding to colibactin-mediated DNA damage and generating colibactin-derived mutational signatures.

Another innovative LC-MS method was developed to measure masses of colibactin DNA-ICL oligonucleotides with sufficient accuracy to determine their molecular formulas, motivating the large-scale production of this species in situ using living bacteria to enable NMR studies. By directly structurally characterizing a colibactin-DNA ICL, we have revealed important information about colibactin. Proposed structures of colibactin, which have been based on biosynthetic knowledge, isolation of decomposition products, and characterization of synthetic analogs, contain ambiguities regarding the identity of the central functional group and the relevance of ring isomers (21,22). We find using both MS and NMR methods only one species bound to DNA that clearly contains an iminium functional group. We hypothesize that the α-ketoimine is derived from oxidation of an initial α-aminoketone or its enolamine tautomer (23,51), though the timing of this oxidation relative to DNA alkylation is unclear. However, the presence of a nitrogen atom-containing central functional group in colibactin was unexpected due to the reactivity of this structural motif and the structures proposed using MS for colibactin and related compounds detected in culture supernatants, which are all suggested to contain a 1,2-diketone. We also see no evidence for DNA alkylation by ring isomers, suggesting that these species, which are generated in the synthesis of stable colibactin mimics and have been observed in decomposition products (24,43), are likely not relevant for colibactin’s DNA damaging activity. While the precise identity of the central functional group initially generated by the colibactin biosynthetic pathway remains elusive, our discovery clearly highlights the relevance of proposed structures containing central nitrogen atoms, and specifically the α-ketoimine, to colibactin-DNA alkylation. It has also been proposed based on the identification of shunt products that the colibactin biosynthetic pathway produces multiple metabolites with the potential to cause DNA damage (52). Our observation of a single major ICL-forming species by MS and NMR calls this into question and suggests there may be additional factors influencing colibactin production and/or stabilization that are still uncharacterized.

Finally, the elucidation of the colibactin-DNA ICL structure reveals how the structural features of colibactin contribute to its DNA damaging activity (Fig. S15). Colibactin can adopt an extended, concave conformation which complements the shape of the narrow, AT-rich minor groove. The heteroaromatic rings flanking the central α-ketoiminium and at the termini of colibactin lie parallel to the walls of the minor groove, enabling hydrophobic and van der Waals interactions. Notably, the orientation of the two thiazole rings reinforces colibactin’s concave shape. The regions that link the central scaffold to the two electrophilic warheads contain multiple methylene groups, derived biosynthetically from glycine and malonyl-CoA (53), making them conformationally flexible and likely facilitating curvature. Colibactin’s positively charged functional groups should also enhance its affinity for DNA. Multiple nitrogen atoms within colibactin form hydrogen bonds and/or electrostatic interactions that influence binding specificity, including the central α-ketoiminium, the amide nitrogens of the two linker regions, and the two pyrrolidinone rings of the electrophilic warheads. Together, these interactions likely position the reactive cyclopropane rings close to N3 of the attacking Ades. This conformation may also be favored by the steric bulk of the pyrroline rings, which are too large to point into the minor groove. Finally, an internal hydrogen bond within the electrophilic warhead between the carbonyl of the pyrrolidinone and a protonated pyrrolinium may be critical for enabling alkylation by enhancing electrophilicity and enabling proper stereochemical alignment of the breaking C─C bond of the spiro-cyclopropane with the extended π-system of the warhead.

Many of the chemical principles likely involved in mediating the specificity of colibactin binding and alkylation (convex shape, aromatic rings, positive charge, hydrogen bond donors) are well-established to be important for AT-rich minor groove recognition by other natural products (54-58), including the polyamide DNA-binding compounds netropsin and distamycin (38), as well as the DNA alkylating duocarmycins, which form monoadducts (59,60). However, colibactin uniquely combines features of AT-rich minor-groove binding specificity with the capacity for interstrand crosslink formation, distinguishing it from other natural products that target DNA. Like other DNA-damaging natural products, the molecular understanding gained in our studies may inspire the design of synthetic agents of therapeutic potential.

Notably, the interaction between the positively charged, central α-ketoiminium of colibactin and the minor groove of DNA is particularly distinctive among AT-rich minor groove binding- and alkylating compounds, which typically contain positively charged functional groups at their termini. The decreased electrostatic potential of the central AATATT motif within the minor groove likely favors binding of the protonated a-ketoiminium of colibactin, contributing to specificity (61). Interestingly, this interaction structurally resembles those of DNA-binding proteins and small molecules, including the positively charged nitrogen atoms of the guanidinium side chain of arginine, which is enriched in proteins that bind AT-rich minor grooves (57) as well as the terminal amidine of the minor groove-binding natural product netropsin (38) (Fig. S22).

The ICL structure, experiments with a synthetic colibactin analog, and calculations highlight the importance of colibactin’s central structural motif for its sequence specificity. Strikingly, the pseudosymmetric structure of colibactin places the positively charged iminium equidistant from the two electrophilic warheads, suggesting it is important for positioning these functional groups. While the instability of the central portion of colibactin motivated the development of a ‘stable’ synthetic colibactin analog lacking this functional group, we find this analog has an altered sequence specificity compared to colibactin produced by *pks*^+^ *E. coli.* This dramatic difference suggests the synthetic analog may not precisely phenocopy the effects of colibactin and calls into question its use as a biologically appropriate surrogate for the natural genotoxin.

In summary, we determined the specificity and structure of the colibactin-DNA ICL, overcoming the challenge of colibactin’s chemical instability by leveraging in situ bacterial production of this genotoxin. Colibactin’s preference for alkylating AT-rich regions matches the locations of colibactin-derived mutational signatures, shedding light on their origins. In particular, our studies help resolve the structure of colibactin’s central region and implicate it as a key determinant of sequence-specific DNA alkylation. This discovery raises the fascinating questions of why such a chemically unstable structural motif would have evolved to play an important role in colibactin’s biological activity and how it is stabilized and/or protected within the cellular environment. Finally, our work highlights innovative experimental strategies that may be used to discover and characterize additional unstable DNA-damaging microbial natural products that may be recalcitrant to traditional isolation approaches.

## Supplementary Material

Supplement 1
