# Supplementary material for: The specificity and structure of DNA crosslinking by the gut bacterial genotoxin colibactin
Source: bioRxiv. 2025 May 27:2025.05.26.655968. Preprint. [Version 1] doi: 10.1101/2025.05.26.655968 (PMC12154741; doi:10.1101/2025.05.26.655968)
Supplement: Supplementary file 1 [file media-1.pdf]

Supporting Information

The specificity and structure of DNA crosslinking by the gut bacterial genotoxin colibactin

Erik S. Carlson[1]†, Raphael Haslecker[2]†, Chiara Lecchi[3], Miguel Aguilar Ramos[1], Vyshnavi Vennelakanti[4,5], Linda Honaker[2], Alessia Stornetta[3], Estela S. Millán[1], Bruce A. Johnson,[6] Heather J. Kulik[4,5], Silvia Balbo[3]*, Peter W. Villalta[3,7]*, Victoria D'Souza[2]*, Emily P. Balskus[1,8]*

Corresponding authors: balskus@chemistry.harvard.edu

The PDF file includes:

Materials and Methods
Supplementary Text
Figs. S1 to S22
Tables S1 to S5
References (*63–91*)

Other Supplementary Materials for this manuscript include the following:

Data S1 – Structural coordinates DFT-optimized structures of colibactin

Materials and Methods

Cell lines and cultures

The *pks*⁻ strain used in this study was *E. coli* BW25113 possessing empty vector (pBeloBAC11) obtained from New England Biolabs. The *pks*⁺ strain was *E. coli* BW25113 possessing pBeloBAC11-*pks* and was a generous gift from the Bonnet Laboratory. Starter cultures of all bacteria were grown overnight aerobically with shaking at 37 ºC in 5 mL of Luria-Bertani (LB-Lennox, RPI) broth containing 34 µg/mL chloramphenicol. Cultures were inoculated from the desired frozen glycerol stock. For all DNA incubation experiments unless stated otherwise, aliquots of each overnight culture were diluted 1:10 in room-temperature Dulbecco's modified Eagle's Medium (DMEM) supplemented with 25 mM HEPES (pH 7.4) and incubated at 37 ºC with constant shaking (190 RPM) until the optical density at 600 nm (OD_{600}) reached ~0.4 – 0.5.

Annealing of synthetic oligonucleotides

Oligonucleotides used in this study were purchased dry from Sigma Aldrich, Integrated DNA Technologies, or Genewiz and reconstituted upon arrival in TE buffer (10 mM Tris-HCl, 1 mM EDTA pH 8.0) to make 100 µM stock solutions. Aliquots of complementary oligonucleotide solutions were combined in a 1:1 equimolar ratio in annealing buffer (10 mM Tris-HCl, 50 mM NaCl, pH 8.0) to make a 10 µM solution. For example, 10 µL of each oligo stock solution were mixed with 80 µL of annealing buffer. The resulting solutions were incubated at 95 ºC for 5 minutes in a heat block, then slowly cooled to room temperature over ~2 h. To verify proper annealing, samples were verified by size using agarose gel electrophoresis. 4% Tris-Acetate-EDTA (TAE) gels were made using NuSieve 3:1 agarose (Lonza) and run at 80 V for 60 min. Gels were pre-stained with Sybr Safe (Thermo Fisher) and visualized with an Azure Biosystems 400 Imager. A table of annealed oligonucleotide substrates is provided in the Supporting Information.

DNA crosslinking assay – bacterial incubations

The protocol was adapted from previously reported assays (*20,24,33*). 2 µg of the desired oligo substrate was diluted to 140 µL of DMEM-HEPES medium in either 1.5 mL Eppendorf tubes or a 96-well culture plate (VWR). DNA solutions were then inoculated with 60 µL of freshly grown bacteria at an OD_{600} = 0.4 – 0.5 (*pks*⁻ or *pks*⁺ *E. coli*; ~20 x 10^6 cells) and incubated at 37 ºC aerobically for 5 h without shaking. If cultured in a 96-well plate, OD_{600} measurements were taken every 5 min to assess growth with brief shaking before each measurement. After this time, the bacteria were pelleted by centrifugation at 10,000 x g for 3 min (4 ºC) and the DNA-containing supernatants were transferred to fresh Eppendorf tubes containing 20 µL of 3 M NaOAc, pH 5. Cell pellets were either saved for *N*-myristoyl-D-asparagine quantitation or discarded. DNA was precipitated by adding 660 µL of cold, 95% EtOH (aqueous, v/v) to the acidified supernatants and storing samples at –20 ºC for ~16 h. The precipitated DNA was isolated by centrifugation at 16,100 x g for 20 min (4 ºC) and removal of the supernatant. The resulting DNA pellet was briefly washed with 200 µL of 70% EtOH (aqueous, v/v) by inversion and re-pelleted by centrifugation at 16,100 x g for 20 min. The supernatant was removed, and the DNA pellet was air dried for ~5 – 10 min before reconstitution in 51 µL of TE buffer. The

concentrations of all DNA samples were determined by analyzing a 1 µL aliquot on a Nanodrop 2000 spectrophotometer (Thermo Fisher). From this point on, all DNA samples were kept on ice while in use or stored at –20 ºC to reduce degradation of colibactin-ICLs.

DNA crosslinking assay – synthetic colibactin analog

The protocol was adapted from previously reported methods and the procedure described above (24). Briefly, 200 ng of the desired oligo substrate was diluted in a citrate buffer (10 mM, pH 5.0) and treated with a mixture of synthetic colibactin analogs (100 µM of a 1:8 mixture of uncyclized and cyclized analogs in DMSO) to a final volume of 20 µL (5% DMSO v/v in water). Assay mixtures were incubated at 37 ºC for 1 hour without shaking and were used immediately for electrophoresis.

Interstrand crosslink analysis by denaturing gel electrophoresis

The protocol was adapted from previously reported methods (*33*). Briefly, 10 µL aliquots of all DNA samples to be analyzed were diluted to 10 ng/µL in TE buffer. DNA concentrations were verified by Nanodrop and adjusted if needed. While on ice, 5 µL of '1% Denaturing Buffer' (1% m/v NaOH, 6% m/v sucrose, 0.01% m/v Orange G) were added to 10 µL of each diluted sample (~100 ng DNA). The denatured samples were then loaded into a 4% Tris-Acetate-EDTA (TAE) gel pre-stained with Sybr Gold (Thermo Fisher). The gel was run at 80 V for 60 min and visualized on an Azure Biosystems 400 Imager. Gel bands were further quantified using ImageJ. Percent crosslinking was calculated by dividing the ICL band intensity by the sum of both ICL and native DNA bands and multiplying by a factor of 100.

DNA strand cleavage assay

Individual oligo substrates were subjected to the DNA crosslinking protocol described above except that the resulting DNA pellets were redissolved in 45 µL of TE buffer. Samples were then heated to 90 ºC for 1 h using a heat block to induce depurination of colibactin ICLs. After samples cooled to room temperature, 5 µL of 3 M NaOAc, pH 5 and 150 µL of cold, 95% EtOH were added in sequence. Samples were stored at –20 ºC overnight to induce DNA precipitation. Precipitated DNA was pelleted by centrifugation (20 min @ 16,100 x g) and the supernatant was removed by pipetting. DNA pellets were briefly air dried (~5 min) and then dissolved in 50 µL of 1 M aqueous piperidine. Samples were heated to 90 ºC for 30 min, cooled to room temperature, and evaporated to dryness using a Labconco Centrivap (45 ºC for ~1 h). The resulting DNA pellets were reconstituted in 51 µL of TE buffer. The sample concentration was determined by Nanodrop analysis. All samples were stored at –20 ºC until further analysis. 5′-FAM-labeled DNA samples were analyzed by sequencing polyacrylamide gel electrophoresis while unlabeled samples were subjected to liquid-chromatography high-resolution accurate-mass mass spectrometry (LC-HRAM-MS).

Analysis of strand cleavage products by sequencing polyacrylamide gel electrophoresis

150 mL of 15% UreaGel solution was prepared by combining SequaGel UreaGel 19:1 denaturing gel reagents (National Diagnostics) according to the manufacturer's protocol.

Polymerization was initiated by adding 60 µL of tetramethylethylenediamine (TEMED; Sigma Aldrich) and 1.2 mL of freshly prepared 10% ammonium persulfate (VWR). This solution was used to cast a 12 in x 16 in x 0.75 mm gel which solidified over 1 h. Prior to sample loading, the gel was pre-run for 30 min at 50 W in Tris-Borate-EDTA (TBE) buffer and all wells were flushed to remove residual urea. Strand cleavage samples were prepared for loading by mixing a 12 µL aliquot (35 ng/µL) with 12 µL of 2X TBE-Urea Sample Buffer (Novex) and heating the mixture to 95 ºC for 5 min. The corresponding A+G Maxam-Gilbert ladders were generated by a previously reported method (*63*). Ladders were diluted to 125 ng/µL and prepared for loading analogously to the strand cleavage samples. After ladder and sample loading, gels were run at a constant 50 W for 4 h. Gel imaging was performed using an Azure Sapphire Imager set for fluorescence detection (ex. 488/ em. 518).

Analysis of intact colibactin-DNA interstrand crosslinks by LC-MS

Sample Preparation

2-3 µg of each of the oligonucleotides was dissolved in 50 µL of 10 mM Tris Buffer containing 1 mM EDTA and transferred to 1.2 mL silanized vials for injection on the LC-MS.

Chromatography for LC-MS.

For each sample, 5 µL were injected onto an UltiMate 3000 RSLCnano UPLC (Thermo Scientific, Waltham, MA) system. Separation was performed using a Shodex HILICpak VN-50 2.0 x 150 mm column (Showa Denko K.K., Tokyo, Japan) maintained at 15 °C using (A) water with 10 mM ammonium acetate and (B) 90:10 acetonitrile:water with 10 mM ammonium acetate. For intact sample analysis, initially 100% B at a flow rate of 200 µL/min was maintained for 6 min followed by linear gradient to 15% B and flow rate to 150 µL/min with a 3 min re-equilibration between injections at the initial conditions. For cleavage samples, initially 100% B at a flow rate of 200 µL/min was maintained for 6 min followed by linear gradient to 40% B over 36 min followed by 0% B over 7 min, with a 4 min hold at 0% B and a 5 min re-equilibration between injections at the initial conditions.

Mass Spectrometry

All mass spectrometric data was acquired with an Orbitrap Lumos mass spectrometer (Thermo Scientific, Waltham, MA). Negative mode electrospray ionization was used with a source voltage of 2.5 kV, a sheath gas setting of 15, and a capillary temperature of 400 °C. Data was collected in profile mode at a resolution setting of 120,000 with a Mass Range setting at High. The S-Lens RF level setting was 60% with full scan detection of m/z 1500-2500 using a normalized AGC Target of 250%, a Maximum Injection Time of 100 ms, and 10 microscans.

Analysis of colibactin-induced strand cleavage products by LC-MS

Sample Preparation

A total amount of 2 µg of each of the cleavage product samples was dissolved in 50 µL of 10 mM Tris Buffer containing 1 mM EDTA and transferred to 1.2 mL silanized vials for injection on the LC-MS.

Chromatography for LC-MS

From each sample 5 µL were injected onto an UltiMate 3000 RSLCnano UPLC (Thermo Scientific, Waltham, MA) system. Separation was performed using a Shodex HILICpak VN-50 2.0 x 150 mm column (Showa Denko K.K., Tokyo, Japan) maintained at 15 °C using (A) water with 10 mM ammonium acetate and (B) 90:10 acetonitrile:water with 10 mM ammonium acetate. For intact sample analysis, initially 100% B at a flow rate of 200 µL/min was maintained for 6 min followed by linear gradient to 15% B and flow rate to 150 µL/min with a 3 min re-equilibration between injections at the initial conditions. For cleavage samples, initially 100% B at a flow rate of 200 µL/min was maintained for 6 min followed by linear gradient to 40% B over 36 min followed by 0% B over 7 min, with a 4 min hold at 0% B and a 5 min re-equilibration between injections at the initial conditions.

Mass Spectrometry

All mass spectrometric data was acquired with an Orbitrap Lumos mass spectrometer (Thermo Scientific, Waltham, MA). Negative mode electrospray ionization was used with a source voltage of 2.5 kV, a sheath gas setting of 15, and a capillary temperature of 400 °C. Data was collected in profile mode at a resolution setting of 120,000 with a Mass Range setting at High. The S-Lens RF level setting was 100% with full scan detection of *m/z* 700-2500 using a normalized AGC Target of 250%, a Maximum Injection Time of 200 ms, and 5 microscans.

Oligonucleotide Data Analysis

Data analysis was performed using Thermo Scientific's Protein Deconvolution and FreeStyle software packages and the online Mongo Oligo Mass Calculator tool.

Cleavage Assay MS Data Analysis

Identifications and relative abundance measurements of the base treatment-induced cleavage sites of the colibactin-exposed double strand oligonucleotides were made using isotopically-resolved charge-state mass deconvolution of their LC-HRAM-MS spectra. The molecular formulas of the base-treatment cleavage products are the same as the "w" and "d" product ions formed upon MS^2 collisional induced dissociation (CID) of negatively charged unmodified single strand oligonucleotides (*64*). The "w" and "d" product ions for each single strand oligonucleotide were calculated online using the Mongo Oligo Mass Calculator v2.06 (http://mass.rega.kuleuven.be/mass/mongo.htm) with the "CID fragments" feature and the "monoisotopic mass", "negative mode", "DNA", and the "5'-OH" and "3'-OH" terminals selected. Mass identities were verified by comparing to calculated masses of proposed structures in ChemDraw. The measured masses of the cleavage products were determined by deconvoluting the multiple charge states seen in full scan spectra acquired during the cleavage product retention period using Freestyle software (Thermo Scientific, Waltham, MA). The

deconvoluted experimental masses from FreeStyle were compared to cleavage products masses calculated using the Mongo software (accounting for the charge state) to assign the cleavage products. The assigned cleavage products were then used to identify the location of the colibactin adduct of the intact oligonucleotide. Signal intensities from both the [M+H] and [M + Na] ions from each cleavage product were summed and normalized to the amount of DNA injected. Values plotted are the difference between the average signal intensities observed in assays with *pks*$^{+}$ and *pks*$^{-}$ *E. coli*.

DNA crosslinking assay in the presence of groove binders

AAATTAATA-50mer (2 µg) was subjected to a modified version of the DNA crosslinking assay conditions described above in which individual DNA groove binding small molecules were added to the assay mixture just before addition of bacteria. Groove binders tested were: netropsin (Enzo Chemicals), DAPI (4′,6-diamidino-2-phenylindole, Sigma Aldrich), methyl green (Chem Impex), and actinomycin D (Acros Organics). Groove binders were added as solutions in DMSO to final concentrations of 0, 5, 10, 50, 100, 500, and 1000 nM while keeping the DMSO concentration ≤ 2% (v/v). The extent of ICL formation was determined by the denaturing gel electrophoresis protocol described above.

Quantitation of *N*-myristoyl-D-asparagine ('prodrug motif')

This assay was adapted from a previous report and was used to confirm production of colibactin in assay mixtures (*65*). Cell pellets obtained from the DNA crosslinking assay were resuspended in 200 µL of LC-MS grade methanol (Honeywell) containing 100 nM d_{27}-*N*-myristoyl-D-asparagine, which served as an internal standard and was prepared as described previously (*34*). Cell suspensions were sonicated for 2 min in a bath sonicator and vigorously vortexed. This was repeated once more before centrifuging the samples at 16,100 x g for 10 min to pellet all cell debris. Sample supernatants were passed through a centrifugal, AcroPrep Advance 96-well 0.2 µM PTFE filter plate (4000 RPM for 10 min, Pall Corp.) and collected in a 96-well clear bottom plate. Samples were either frozen at –20 ºC to prevent evaporation or immediately analyzed by a previously reported liquid chromatography negative electrospray-ionization tandem mass spectrometry (UPLC-ESI^{-}-MS/MS) method.

Liquid chromatography was performed using a Waters Acquity UPLC H-Class System (Waters Corporation) equipped with an Agilent Poroshell 120 EC-C18, 2.7 µm, 4.7 mm x 50 mm column using a multistep gradient. Conditions started at 10% solvent B at 650 µL/min for 0.5 min, followed by a linear gradient to 95% B over 0.5 min, a hold at 95% B for 1 min, and a linear gradient back to 10% B over 0.5 min where the column re-equilibrated for 1 min (solvent A, 95:5 water/methanol + 0.03% NH_4OH; solvent B, 80:15:5 isopropanol/methanol/water; injection volume = 5 µL). Mass spectrometry was performed using a Waters Xevo TQ-S UPLC-triple quadrupole mass spectrometer. The multi-reaction monitoring (MRM) transitions were *m/z* 341.3 → *m/z* 226.3 (collision energy (CE), 24 V; cone voltage, 50) for unlabeled prodrug motif and *m/z* 368.5 → *m/z* 253.3 (CE, 28 V; cone voltage, 58 V) for D_{27}-prodrug motif. Data analysis was conducted using TargetLynx software. Unlabeled prodrug concentrations were calculated by converting the peak area ratios (unlabeled prodrug/d_{27}-prodrug) to concentration ratios using a

freshly run calibration curve of varying unlabeled prodrug containing 100 nM d_{27}-prodrug and multiplying by internal standard concentration (100 nM).

Large-scale production of a colibactin-DNA ICL for NMR spectroscopy

~192 µg of double-stranded 2′-fluoro-14mer (Genewiz) were diluted in 13.5 mL of DMEM-HEPES. The resulting solution was dispensed into a 96-well plate in 140 µL aliquots and subjected to the DNA crosslinking assay described above except incubations were performed for 16 h at 30 ºC. Two columns of the 96-well plate were used as negative controls (media blank and *pks*$^{-}$ *E. coli*); all other columns contained *pks*$^{+}$ *E. coli*. After the incubation, half of the wells in each column were combined to give two samples per column (24 samples total, ~800 µL) which were centrifuged at 10,000 x g for 5 min to pellet cells. The supernatants were then divided into two 400 µL aliquots and each aliquot was treated with 40 µL of 3M NaOAc, pH 5 and added to 880 µL of 95% EtOH (aqueous, v/v) to precipitate DNA. Samples were stored at –20 ºC overnight.

DNA-precipitated samples were centrifuged at 16,100 x g for 30 min (4 ºC) to pellet DNA. The supernatants were removed, and each DNA pellet was washed with 200 µL of 70% EtOH (aqueous, v/v). The DNA was re-pelleted by centrifugation (16,100 x g for 15 min) and the supernatant was removed. The DNA pellets were air-dried for ~5 min. Samples originating from the same column on the 96-well plate were reconstituted in 125 µL of TE buffer and pooled to make 12 samples (500 µL total volume). The extent of ICL formation in each sample was checked using the previously denaturing gel electrophoresis method. Finally, *pks*$^{+}$ samples were pooled, desalted with water and concentrated to ~50 µL using Amicon Ultra 3K – 0.5 mL spin filters (16,100 x g for 30 min, Millipore) and pooled.

This entire protocol was repeated twice to generate a total of ~410 µg of partially crosslinked DNA from 3 96-well plates. All DNA from *pks*$^{+}$ samples was combined to generate the final sample, which was stored at –20 ºC until further processing and analysis by NMR spectroscopy.

Production of a [^{15}N,^{13}C]-colibactin-DNA ICL for NMR spectroscopy

DNA containing an ICL with isotopically labeled colibactin was produced analogously to the unlabeled sample except all incubations were performed in M9 minimal medium containing [^{15}N]-ammonium chloride (99%, Cambridge Isotope Laboratories, Inc.) and [$^{13}C_6$]-D-glucose (99%, Cambridge Isotope Laboratories, Inc.). Additionally, overnight starter cultures were sub-cultured in this isotopically labeled medium instead of DMEM-HEPES. Isotope incorporation was assessed by mass spectrometry using the protocol described above for intact colibactin-DNA interstrand crosslinks and revealed >90% isotope incorporation.

NMR spectroscopy

CYANA library residue design

As no CYANA library residue for a colibactin modified base currently exists, it had to be constructed anew. The colibactin molecule was drawn using ChemDraw 21.0.0 according to the

structure inferred from the analysis of intact colibactin-DNA interstrand crosslinks described above and knowledge from prior studies (*21,22*). An adenosine monophosphate residue was N3-linked onto the opened warhead to mimic a monoalkylated residue on the imino-facing side of colibactin. The opposite end of the colibactin molecule had a single methyl group of the opened warhead removed. This was necessary, as CYANA is unable to model the interstrand crosslinked adenosines as a single molecule in the context of any DNA or RNA chain. Thus, in order to accurately represent the link, an N3-methyladenosine residue was also constructed using ChemDraw, representing the colibactin modified base on the ketone-facing side. The ChemDraw structures were transformed to PDB files using OPENBABEL's cdxml to pdb function (*66*).

The exported file was then manually adjusted to fit the CYANA library format. A custom R script was then used to center the coordinates according to CYANA specifications and rearrange the atom order to the default of adenosines in the CYANA library such that all bonding and dihedral parameters specify the correct atoms on the new base. Dihedral parameters for the adenosine atoms were copied from standard adenosine residues, all colibactin dihedral angles were manually specified by their degrees of freedom according to the known stereochemistry, sp-hybridization, and aromaticity. Finally, for CYANA calculations, a bond between the warhead-methyl end of colibactin and the N3-methyl of the opposite strand A+3 residue of length 1.48-1.52 Å was included, thereby mimicking the complete colibactin ICL.

Xplor library residue design

For Xplor, the topology and parameter file templates were generated using the ChemDraw structures and PRODRG version AA100323.0717 (*67*). The atom parameters and topology values in the exported templates were manually adjusted according to the stereochemistry of proposed colibactin structures as determined by prior biosynthetic and synthetic studies. The pyrrolidinone, pyrroline, and thiazole ring parameters were adjusted according to the following references, respectively (*68-70*). Planarity, angles, and dihedrals were adjusted according to the stereochemistry of proposed colibactin structures as determined by prior biosynthetic and synthetic studies. Even calculations where these angles were freely rotated converged with prior stereochemistry. The end connectors of colibactin and the N3-methyladenosine were also added manually and their repulsion lowered to comply with the features of the covalent C–C bond. The N3-methyl residue on the connecting adenosine was written and adjusted manually. Similarly, for cases in which the structural features were not known, they were modeled manually, such as the axial vs equatorial positions of the terminal pyrrolidines, the single or double protonation of the α-iminoketone and the *cis*- vs *trans*-orientation of the thiazole rings, the parameter and topology files were adjusted manually.

NMR data acquisition and resonance assignment

DNA samples were suspended in buffer (10 mM Tris-HCl pH 8.2, 10mM NaCl) by washing them five times using Amicon centrifugal filters. All NMR experiments were acquired in 5 mm Shigemi tubes with a Bruker 800 MHz instrument containing a cryogenic probe. Spectra for observing non-exchangeable protons were collected in 100% D_2O at 298 ºK and for exchangeable protons in 90% H_2O an at 278 ºK. ^1H-^1H NOESY and TOCSY were recorded with

unlabeled samples and ^{15}N-HSQC spectra were collected by using ^{13}C^{15}N-labeled samples. All data was analyzed using NMRDraw v11.1, NMRviewJ 8.0.3, and NMRFx Analyst v11.2.4-c.

Structural modeling

Initial structural models were generated using manually assigned restraints in CYANA, where upper-limit distance restraints of 2.7, 3.3, and 5.0 Å were employed for direct NOE cross-peaks of a strong, medium, and weak intensities, respectively (*71*). To prevent the generation of structures with collapsed major grooves, cross-helix P–P distance restraints (with 20% weighting coefficient) were employed for B-form helical segments. Standard torsion angle restraints were used for the B-helical geometry, allowing for ±50° deviations from ideality ($\alpha = -68°$, $\beta = -147°$, $\gamma = 46°$, $\delta = 135°$, $\varepsilon = -150°$, $\zeta = -100°$) (*72*). Standard hydrogen-bonding restraints with approximately linear NH–N and NH–O bond distances of 1.9 ± 0.1 Å and N–N and N–O bond distances of 2.9 ± 0.01 Å.

The CYANA structure with the lowest target function was used as the initial model for structure calculations Xplor-NIH to incorporate electrostatic constraints. First, structures were calculated using annealing from 2000 °C to 25 °C in steps of 12.5 °C. Standard energy potential terms for bonds, angles, torsion angles, van der Waals interactions, and interatomic repulsions were included. Energy potentials for NOEs, hydrogen bonds, and planarity were incorporated with restraints derived from NMR data. All restraints used in CYANA were included except for phosphate-phosphate distances. The structures were sorted by energy using bond, angle, dihedral, and NOE energy potential terms and the ten percent of the structures with the lowest sort energy. The lowest ten percent of these were deposited in the RCSB data bank.

Structure deposition

NMRFx Analyst was used to confirm distance restraints used for the structure calculations and generate NMR-STAR format files for uploading to the BMRB. To do this, an N3-methyladenosine residue with a fluorine at the F2' position was constructed for the NMRFx residue library. This allowed loading in the DNA sequence with the modified residue in each of the two strands. A PDB file, containing the atoms specific to the colibactin molecule was extracted from the file generated by XPLOR. This PDB file was read to load in the colibactin molecule to form the complete complex. Next, a peak list file (in NMRFX .xpk2 format) was loaded containing NOE cross peaks. This peak list was used to populate a table of distance restraints and violations (given the loaded 3D structure). The molecular viewer in NMRFx was also used to visualize constraints in the context of the 3D structure. NMRFx was then used to export the NMR-STAR file containing the molecular assembly and chemical shift assignments.

<u>Computational Modeling</u>

Calculations of colibactin electrostatic potential

We computed the electrostatic potential (ESP) of specific atoms on proposed colibactin structures and doubly charged DNA with sequences 5'-GAATATTC-3' and 5'-GAACGTTC-3'

following previously reported protocols (*73-75*). We computed the partial charges on atoms of interest using iterative Hirshfield (Hirshfield-I) charges (*76,77*) as implemented in Multiwfn version 3.7 (*78*) and applied these charges to compute the ESP at these atoms (*75*). All ESP values were obtained from geometry optimization calculations carried out with density functional theory (DFT) at the B3LYP (*79-81*) -D3 (*82*) /6-31G* (*83–86*) level of theory.

Calculation of DNA electrostatic potential

We used the online tool DNAphi provided by the Rohs lab (https://rohslab.usc.edu/DNAphi/index.html) (*87*) which predicts ESP of minor grooves by solving the non-linear Poisson-Boltzmann equation of DNA fragments.

Modeling of colibactin ICLs containing alternative central base pairs

We used the restraints generated via NMR (see above) and substituted the central or flanking base pairs for generating initial models of non-ideal motifs crosslinked to colibactin by CYANA followed by the introduction of electrostatics by Xplor as described above.

Synthesis of synthetic colibactin analog

Intermediates **S3** (*24*) and **S5** (*22*) were prepared according to published procedures. The route to obtain **S4** was modified from previous reports (*24*), and **S4** was used to access the stable colibactin analog and cyclized diastereomers as an inseparable 1:8 mixture as previously described (*24*).

Proton nuclear magnetic resonance (^{1}H NMR) and proton-decoupled carbon nuclear magnetic resonance (^{13}C NMR) spectra were recorded at 400 or 101 megahertz (MHz), respectively, on a Bruker Avance NEO. Proton chemical shifts are expressed in parts per million (ppm, δ scale) and are referenced to residual protium in the NMR solvent ($CHCl_3$: δ 7.26 or $CHDCl_2$: δ 5.32). Carbon chemical shifts are expressed in parts per million (ppm, δ scale) and are referenced to the carbon resonance of the NMR solvent ($CHCl_3$: δ 77.2 or $CHDCl_2$: δ 53.8). Data are represented as follows: chemical shift, multiplicity (s = singlet, d = doublet, t = triplet, br = broad), coupling constant (J) in Hertz (Hz) and integration. For LC–HRMS analysis, an Agilent Q-TOF 6530 equipped with a Dual AJS ESI source in positive mode with a Dikma Inspire C18 column (5µm, 5 x 4.6mm) was used. Solution A was H_2O + 0.1% formic acid and solution B was acetonitrile + 0.1% formic acid. The LC method was: 1 min at 5% Solution B, 4 min for 5 to 95% Solution B, 1.5 min at 95% Solution B, 0.5 min for 95% to 5% Solution B, and 2 min at 95% Solution B with a flow rate of 0.5 mL/min. The following parameters were used for the Q-TOF: Gas Temperature 300 ºC, Drying Gas 11 L/min, Nebulizer 35 psi, Sheath Gas Temperature 275 ºC, Sheath Gas Flow 11 L/min, VCap 3500 V, Nozzle Voltage 500 V.

Synthetic route to the stable colibactin analog

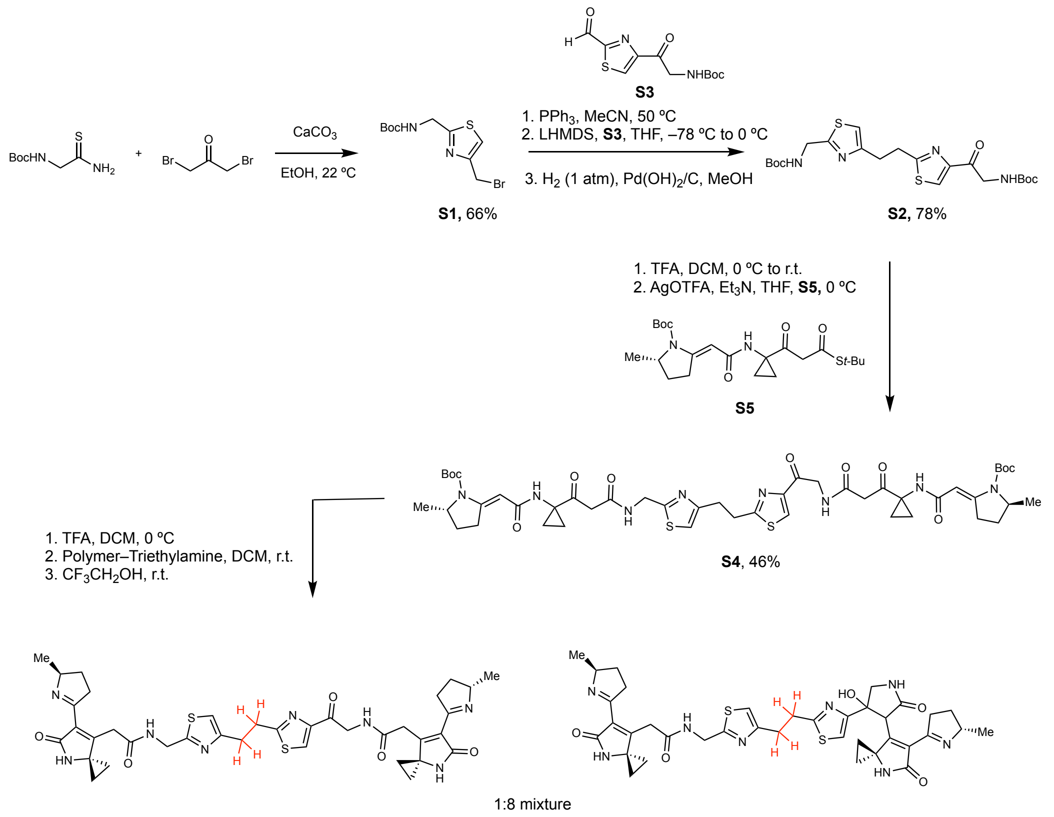

*Preparation of bromide **S1***

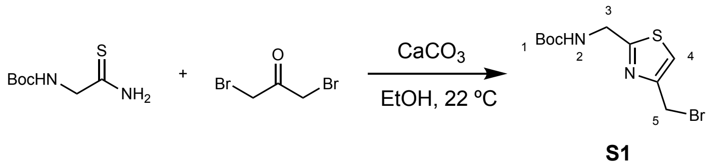

Reaction was based on a previously reported procedure (*24*). *tert*-Butyl (2-amino-2-thioxoethyl)carbamate (40.0 mmol, 1.0 equiv) was dissolved in anhydrous ethanol (300 mL). To this solution, calcium carbonate (240.0 mmol, 6.0 equiv) and 1,3-dibromopropan-2-one (52.0 mmol, 1.30 equiv) were added and stirred at room temperature for 18 hours. The reaction mixture was filtered through a celite pad and washed with ethyl acetate (50 mL). The filtrate was combined, concentrated, and redissolved in ethyl acetate (350 mL) and extracted with a saturated sodium bicarbonate solution (2 x 75 mL) and brine (1 x 50mL). The organic layer was dried over sodium sulfate, filtered, and concentrated. The residue obtained was purified using automated flash-column chromatography (Biotage Selekt) (10% to 50% EtOAc in Hexanes to afford the title product as a light tan solid (8.12 g, 66% yield). R_f = 0.53 (50% EtOAc in Hexanes, UV lamp). ^{1}H NMR: (400 MHz, $CDCl_3$) δ(ppm) = 7.23 (s, 1H, H_4), 5.32 (br, 1H, H_2), 4.59 (d, J = 6.2 Hz, 2H, H_3), 4.54 (s, 2H, H_5), 1.46 (s, 9H, H_1). ^{13}C NMR: (101 MHz, $CDCl_3$) δ(ppm) = 170.09, 155.74, 151.95, 118.15, 80.46, 42.55, 28.45, 27.11. HRMS(ESI-AJS): calc'd for $C_{10}H_{16}BrN_2O_2S^+$ [M+H]$^+$, 307.0110; found, 307.0112.

*Preparation of bisthiazole **S2***

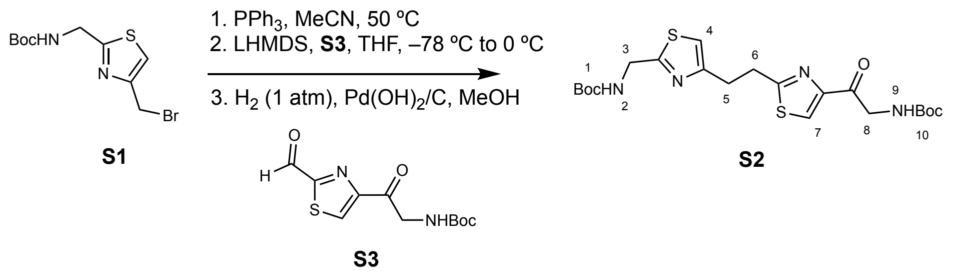

Reactions were based on a previously reported procedure (*24*). Bromide **S1** (5.50 mmol, 1.00 equiv) was dissolved in anhydrous acetonitrile (20 mL). Triphenylphosphine (6.05 mmol, 1.10 equiv) was added and the mixture heated to 50 ºC for two hours. The mixture was concentrated under vacuum and triturated with ether to afford a phosphonium bromide which was used directly in the next step.

The phosphonium bromide (3.30 mmol, 1.10 equiv) was dissolved in anhydrous THF (100 mL) and cooled to –78 ºC in a dry ice/acetone bath. LHMDS (1 M in THF, 3.60 mmol, 1.20 equiv) was added dropwise and the resulting mixture was stirred for 15 minutes at –78 ºC. A solution of **S3** (3.00 mmol, 1.00 equiv) dissolved in THF (20 mL) was added dropwise over 15 minutes. The reaction mixture was stirred for 30 minutes at –78 ºC and warmed to 0 ºC over 45 minutes. The reaction mixture was then diluted with saturated sodium bicarbonate (20 mL), water (20 mL), and ethyl acetate (50 mL). The aqueous layer was extracted with ethyl acetate (2 x 40 mL) and the combined organic layers were washed with brine (1 x 30 mL), dried over sodium sulfate, filtered, and concentrated. The crude mixture was resuspended in methanol (50 mL) and $Pd(OH)_2$/C (20 mg) was added to the suspension. The flask was evacuated, refilled with H_2 (1 atm, balloon), and left to react overnight. The resulting mixture was filtered through celite, and the filter cake was washed with methanol (2 x 30 mL). The filtrate was concentrated, and the residue obtained was purified using automated flash-column chromatography (30% to 60% EtOAc in Hexanes) to afford the title product as a white solid (113 mg, 78% yield). R_f = 0.14 (50% EtOAc in Hexanes, UV lamp) ^{1}H NMR: (400 MHz, CD_2Cl_2) δ(ppm) = 8.07 (s, 1H, H_7), 6.88 (s, 1H, H_4), 5.55 (s, 1H, H_2), 5.38 (s, 1H, H_9), 4.60 (d, *J* = 5.1 Hz, 2H, H_8), 4.56 (d, *J* = 6.1 Hz, 2H, H_3), 3.45 (t, *J* = 7.7 Hz, 2H, H_6), 3.24 (t, *J* = 7.5 Hz, 2H, H_5), 1.45 (s, 9H, H_1 or H_{10}), 1.44 (s, 9H, H_1 or H_{10}). ^{13}C NMR: (101 MHz, CD_2Cl_2) δ(ppm) = 190.28, 170.88, 169.69, 156.05, 156.01, 155.07, 152.47, 125.89, 114.72, 80.17, 79.80, 49.39, 42.81, 33.08, 31.39, 28.48, 28.46. HRMS(ESI-AJS): calc'd for $C_{21}H_{31}N_4O_5S_2^+$ $[M+H]^+$, 483.1730; found, 483.1734.

*Preparation of imide **S4** (24)*

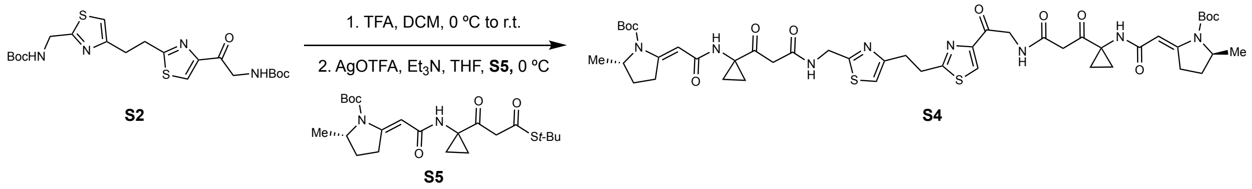

Reaction was based on a previously reported procedure (*24*). The bisthiazole **S2** (0.15 mmol, 1.00 equiv) was dissolved in anhydrous DCM (3 mL) and cooled to 0 ºC in an ice bath. Neat trifluoroacetic acid (7.50 mmol, 50.0 equiv) was added dropwise, and the resulting mixture stirred for three hours at room temperature. The solvent was removed under a stream of argon, and the residue was resuspended in 1 mL of DCM and solvent was removed again. The resulting residue was dried under high vacuum and used immediately in the next step.
Thioester **S5** (0.33 mmol, 2.20 equiv) was added to the flask containing the bisamine salt, and both reagents were dissolved in anhydrous THF (2 mL). Triethylamine (1.20 mmol, 8.00 equiv) was added dropwise and the mixture was covered with aluminum foil. Silver triflate (0.39 mmol, 2.60 equiv) was dissolved in THF (1 mL) and this solution was added dropwise to the amine mixture. The mixture was stirred for 30 minutes at 0 ºC before quenching with ethyl acetate (20 mL) and saturated aqueous ammonium chloride (10 mL). The mixture was filtered through a celite pad and rinsed with ethyl acetate (10 mL). The layers were separated, and the aqueous layer was further extracted with ethyl acetate (1 x 10 mL). The combined organic fractions were washed with brine (1 x 20 mL) and the organic layer was dried over sodium sulfate, filtered, and concentrated. The product was purified by automated flash-column chromatography (EtOAc to 20% isopropanol in EtOAc) to afford the title product as a white solid (67 mg, 46% yield). R_f = 0.24 (5% MeOH in DCM, UV lamp). ^{1}H and ^{13}C NMR spectra matched those previously reported (*24*). HRMS(ESI-AJS): calc'd for $C_{47}H_{63}N_8O_{11}S_2^+$ [M+H]$^+$,979.4052; found, 979.4047.

Supplementary Text

Discussion of intact ICL oligonucleotide MS data analysis

The 14mer and 25mer dsODN substrates were characterized using LC-HRAM-MS after *pks*$^+$ *E. coli* incubation. For each dsODN that became crosslinked, we observed a mixture of unmodified and modified charge states which were deconvoluted to attain their molecular masses using the deconvolution feature of the FreeStyle software tool (Thermo Scientific, Waltham, MA). The mass difference between the modified and colibactin-adducted oligonucleotides was used to differentiate between the molecular formulas of the two proposed colibactin crosslinking structures, the α-ketoimine and diketone structures. The analysis of the unmodified 14mer dsODN (5′-CGCGAAATTTCGCG-3′; Fig. 3) provided an accurate mass determination of 8523.51 Da (actual: 8523.49 Da). The measured mass of the modified dsODN was 9294.77 Da with a mass difference (ΔM) of 771.26 Da from the unmodified dsODN, which is consistent with the proposed α-ketoimine crosslink structure, rather than the alternative diketone crosslink structure (ΔM = 772.24 Da). This type of analysis performed on 25mer dsODNs resulted in a greater uncertainty in the mass difference measurement (ΔM = 771±1 Da) due primarily to the reduced relative intensity of the monoisotopic peak of the modified dsODN and the difficulty in identifying the monoisotope of the crosslinked dsODN among the baseline signal of unresolved background signal at each nominal mass. An analysis of the selected ion mass ranges of 2185 - 2189 *m/z* and 2295 - 2299 *m/z* for detection of the 7- charge state of the unmodified 5′-GATCAAGCGAATATTATACGACTCA-3′ and 3′-CTAGTTCGCTTATAATATGCTGAGT-5′ dsODN ($C_{491}H_{618}N_{185}O_{296}P_{48}$) and the colibactin-modified dsODN, respectively, was performed. The molecular formulas of the unmodified, proposed α- ketoimine-crosslinked and the proposed diketone-crosslinked dsODN are $C_{491}H_{618}N_{184}O_{296}P_{48}$ (Fig. S12A), $C_{528}H_{659}N_{193}O_{302}P_{48}S_2$ (Fig. S12B) and $C_{528}H_{658}N_{192}O_{303}P_{48}S_2$ (Fig. S12C), respectively. Figure S12 shows that the simulated

isotopic patterns for the unmodified dsODN (A) and the α-ketoimine-crosslinked dsODN structure (B) match the experimental spectra whereas the diketone-crosslinked dsODN structure (C) shows a slight but clear offset. This is consistent with our assignment of the α-ketoimine-crosslinked dsODN structure using the experimental data for the 14mer dsODN analysis as discussed above and in the main body of the manuscript.

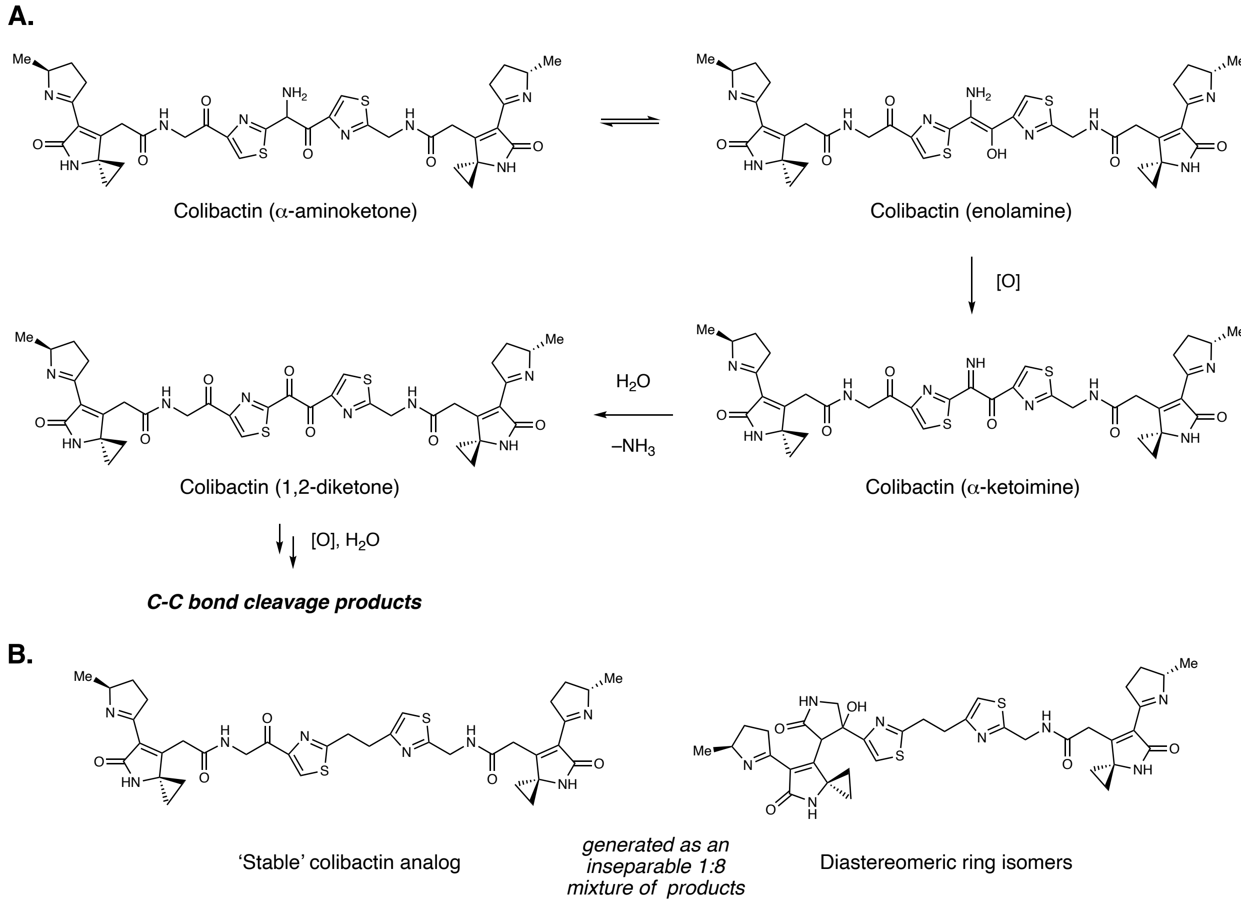

Fig. S1.
Chemical structures of colibactin and synthetic colibactin analogs. (A) The central region of the proposed chemical structure of colibactin is proposed to undergo oxidative decomposition. (B) The chemical structures of the synthetic 'stable' colibactin analog and inseparable diastereomeric cyclized products.

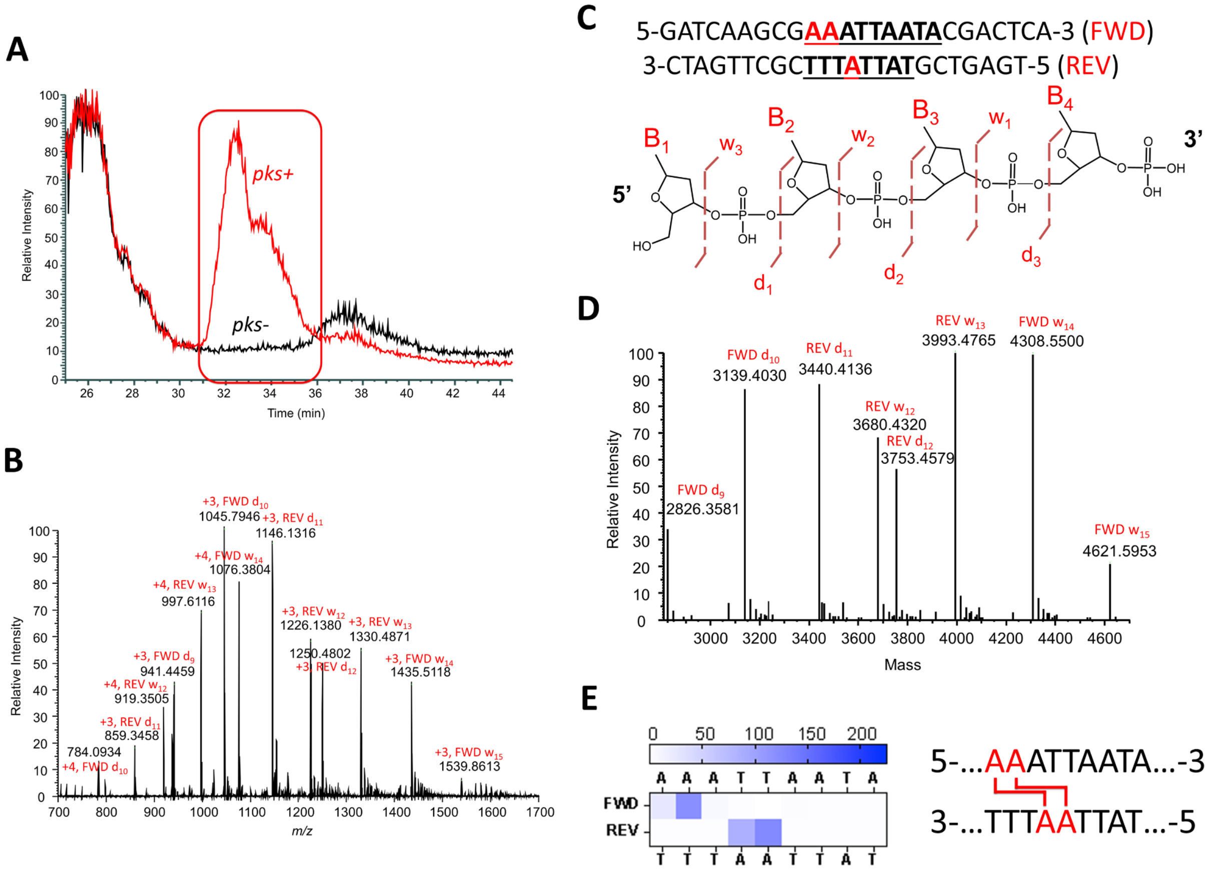

Fig. S2.

LC-MS cleavage analysis of crosslinked 25-mer DNA double strand oligonucleotide (5'-GATCAAGCGAAATTAATACGACTCA-3') exposed to *pks*$^+$ *E. coli.* (A) Total ion chromatograms of *pks*$^+$ and *pks*$^-$ *E. coli*-treated samples (B) Full scan spectrum (retention time = 31 - 36 min) of the *pks*$^+$ *E. coli* treated sample with assigned ion peaks. (C) Double strand oligonucleotide and illustration of cleavage fragmentation process (D) Deconvoluted spectrum of the full scan ion signal in (B). (E) Illustration of interstrand crosslinking locations and heat map of abundance of crosslinking.

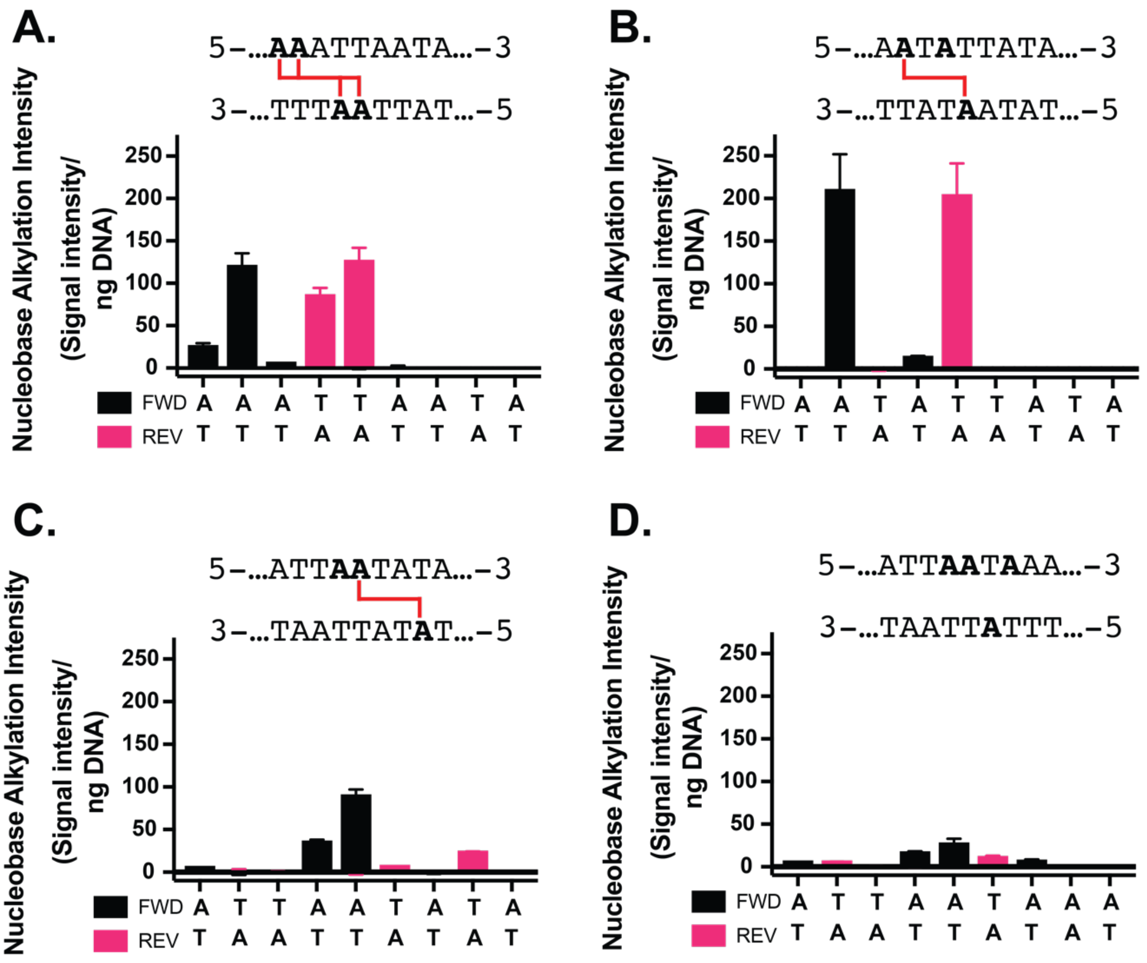

Fig. S3.

LC-MS strand cleavage data shown as column graphs. Y-axes represent the mass spec signal intensity of the cleavage product ions produced by cleavage (crosslinking) at the position of the oligo strand shown in the x-axis. Intensities are the difference of *pks*$^{+}$ and *pks*$^{-}$ *E. coli*-treated samples normalized to the amount of DNA injected. Error bars represent the mean +/– the s. d. of three biological replicates.

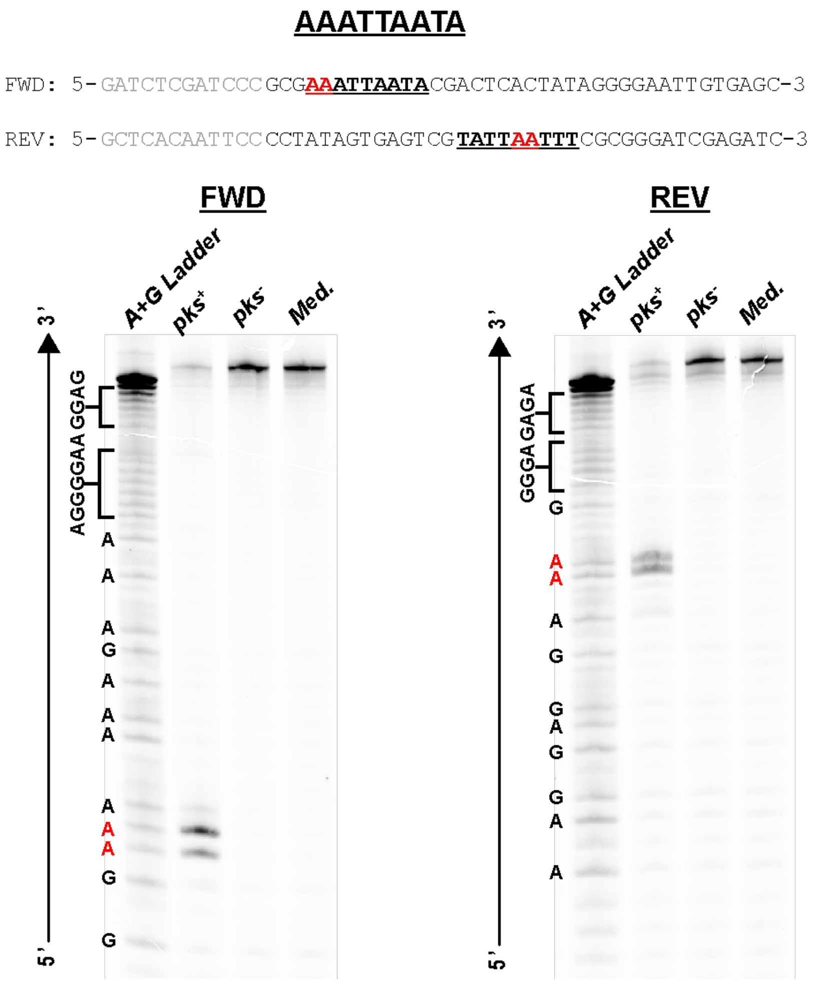

Fig. S4.
Sequencing gel of AAATTAATA-derived strand cleavage products after exposure to *pks*+ *E. coli.* Both the forward (left) and reverse (right) strands are independently labeled with 5′-FAM. Exposure of the DNA to *pks*- *E. coli* and uninoculated media (Med.) serve as negative controls.

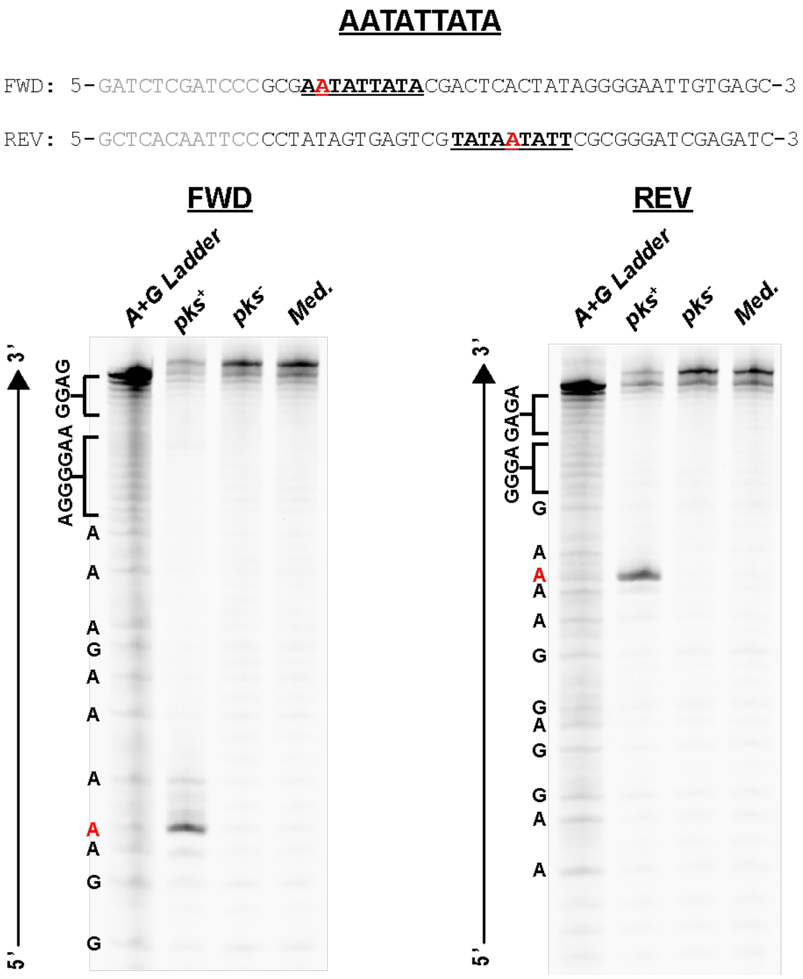

Fig. S5.
Sequencing gel of AATATTATA-derived strand cleavage products. after exposure to *pks*$^{+}$ *E. coli.* Both the forward (left) and reverse (right) strands are independently labeled with 5′-FAM. Exposure of the DNA to *pks*$^{-}$ *E. coli* and uninoculated media (Med.) serve as negative controls.

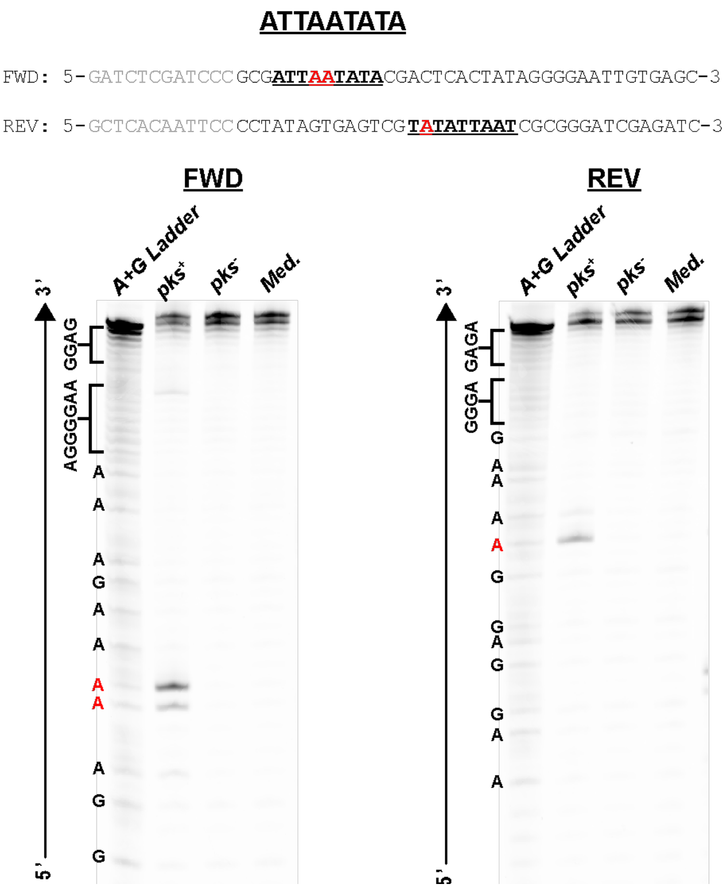

Fig. S6.

Sequencing gel of ATTAATATA-derived strand cleavage products after exposure to *pks*+ *E. coli*. Both the forward (left) and reverse (right) strands are independently labeled with 5′-FAM. Exposure of the DNA to *pks*- *E. coli* and uninoculated media (Med.) serve as negative controls.

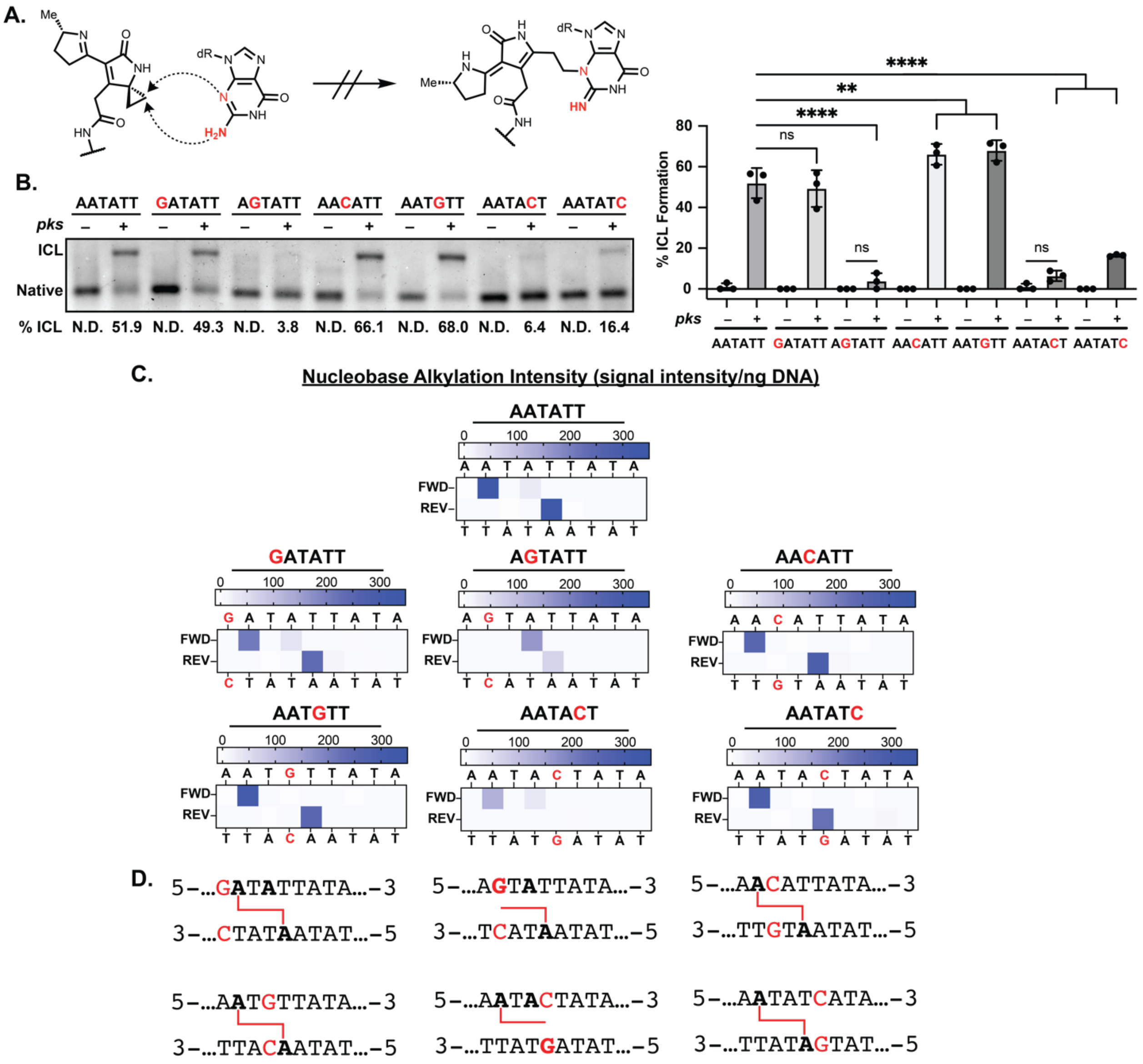

Fig. S7.

Colibactin does not alkylate guanine but can form adenine monoadducts. (A) Scheme depicting the possible reactivity of colibactin with guanine. (B) Denaturing gel analysis showing DNA interstrand crosslinking of 50mers containing sequence variants of 5'-AATATT-3'. Mean values of ICL formation are provided below. Statistical analyses are shown in a column plot. (C) Residue-specific alkylation of 25mers containing the indicated sequence variants of 5'-AATATT-3'. Alkylation intensities were determined through liquid chromatography-high resolution accurate mass-mass spectrometry (LC-HRAM-MS) analysis and normalized to total DNA injected. Intensity reported is difference between the average detected in assays with *pks*$^{+}$ *E. coli* compared to the average detected in assays with *pks*$^{-}$ *E. coli*. (D) Inferred ICL and monoadduct locations within tested sequence motifs. All alkylated residues are bolded. ICLs and monoadducts are represented by red lines. Data are mean +/- s.d. with n=3 replicates. ****P < 0.0001; **P < 0.01; ns (not significant) P > 0.05, one-way ANOVA and Tukey's multiple comparison test.

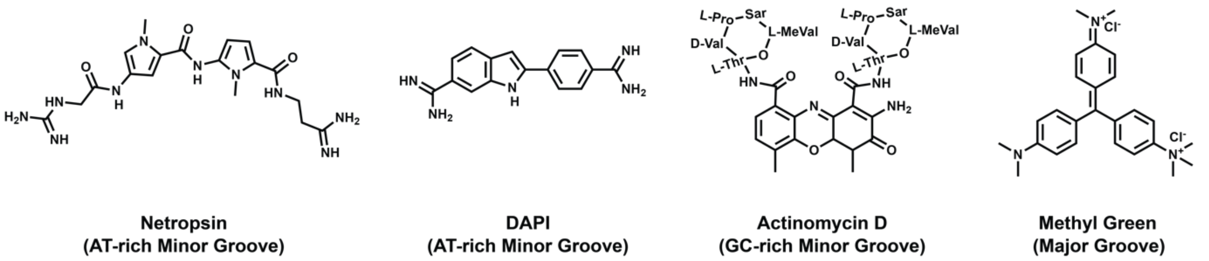

Fig. S8.
Chemical structures and specificities of DNA groove binders used in this study.

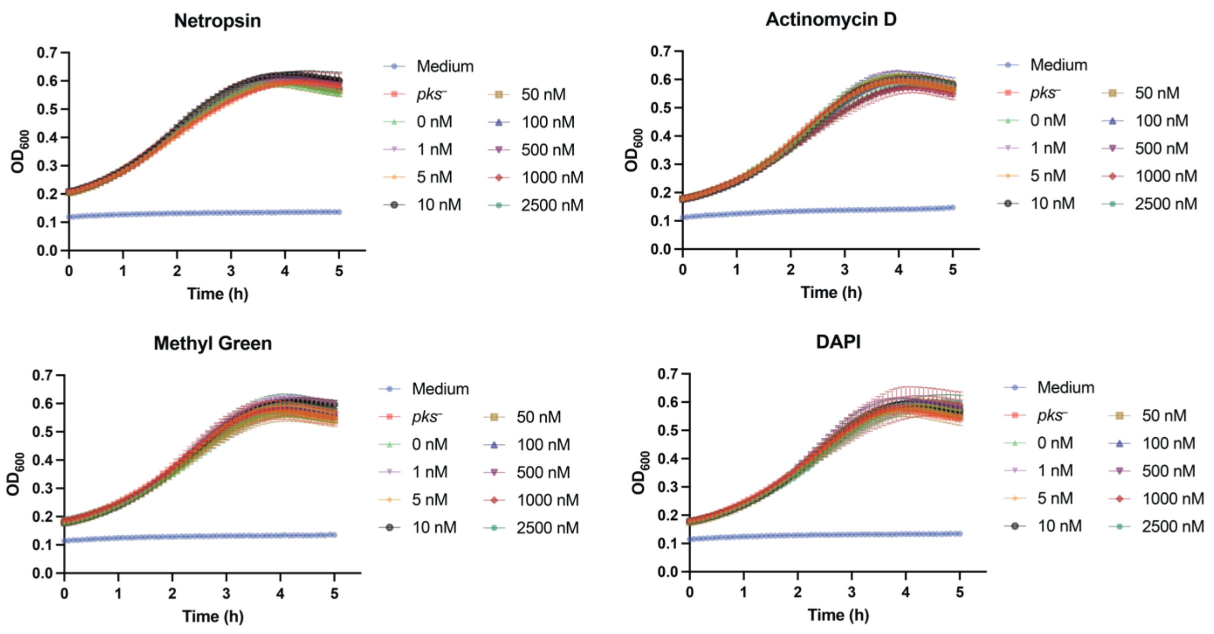

Fig. S9.

Growth curves for *E. coli* in the presence of DNA groove binders. Growth was monitored at by measuring the optical density at 600 nm (OD_{600}). Data are mean ± s.d. ($n = 3$).

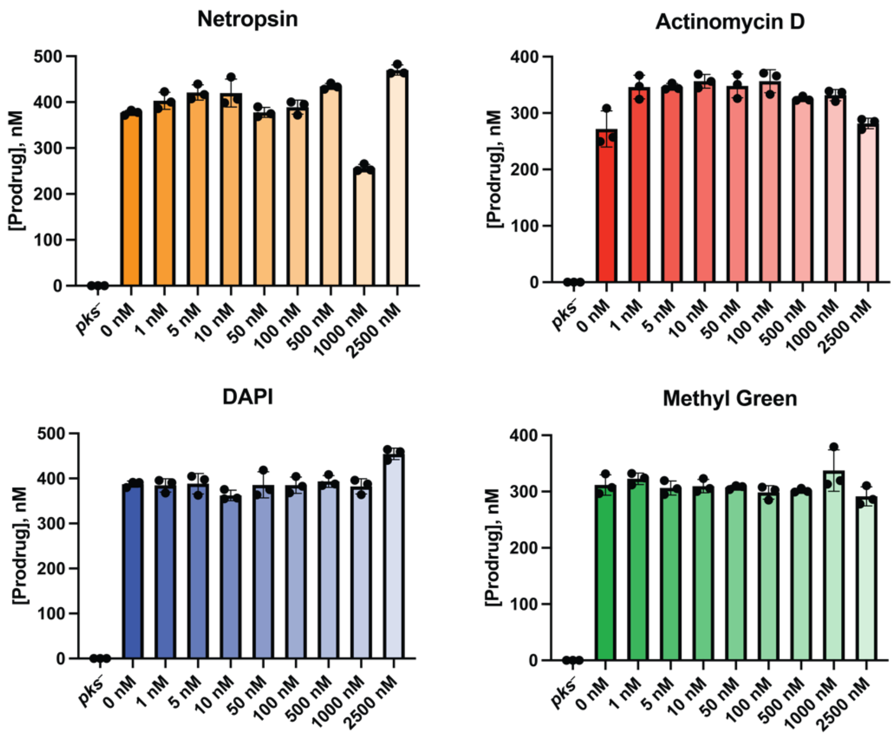

Fig. S10.
N-myristoyl-ᴅ-asparagine (prodrug) quantitation by liquid chromatography negative electrospray ionization tandem mass spectrometry (LC-ESI⁻-MS/MS) shows production of colibactin by *pks*⁺ *E. coli* during growth in the presence of varying concentrations of DNA groove binders. Data are mean ± s.d. (n = 3).

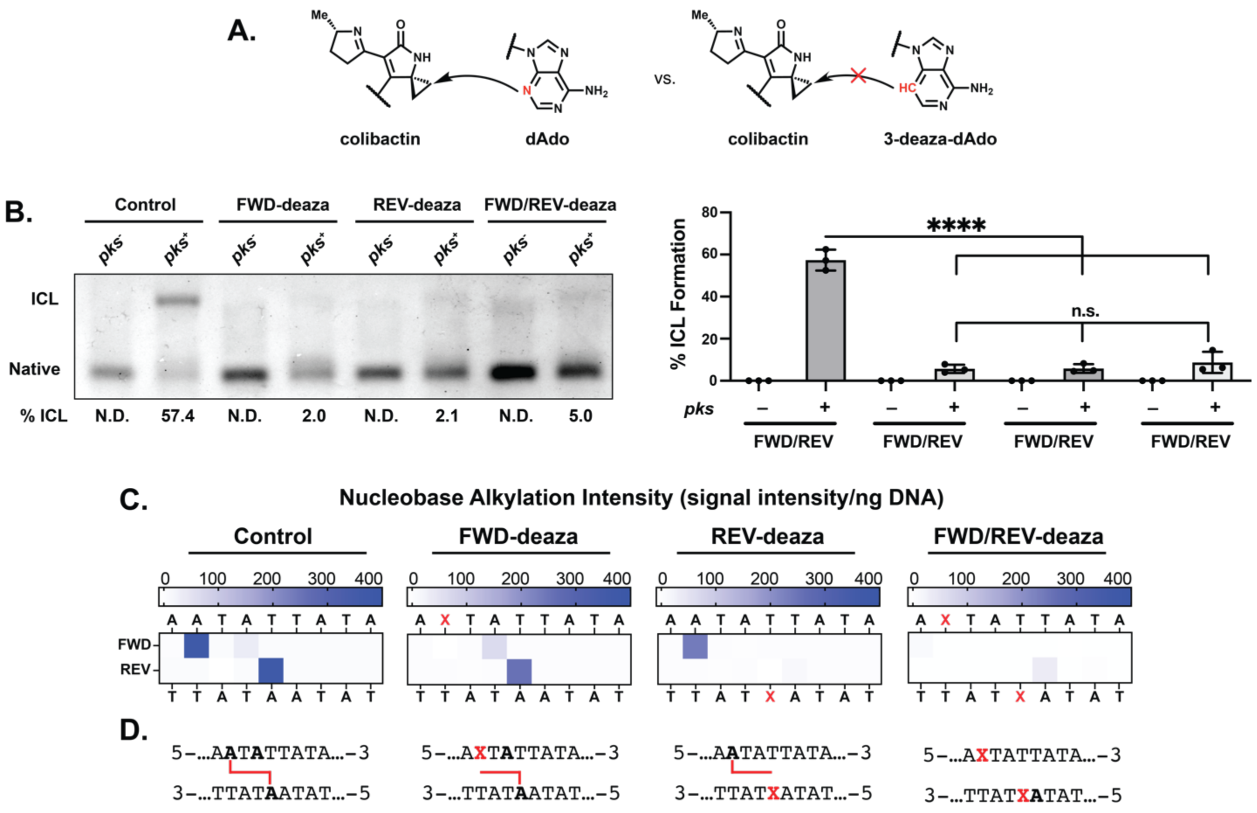

Fig. S11.

Colibactin exclusively alkylates the N3-position of adenine. (A) Scheme depicting site-selective incorporation of 3-deaza-dAdo inhibiting DNA alkylation and ICL formation by colibactin. (C) Denaturing gel analysis of DNA interstrand crosslinking within 50mers containing N3-deaza-dAdo-sequence variants of 5'-AATATTATA-3'. (C) Levels of site-specific nucleobase alkylation within 25 bp sequence analogs of above sequences. Alkylation intensities were determined through liquid chromatography-high resolution accurate mass-mass spectrometry (LC-HRAM-MS) analysis and normalized to total DNA injected. Intensity reported is difference between the average detected in assays with *pks*$^{+}$ *E. coli* compared to the average detected in assays with *pks*$^{-}$ *E. coli*. (D) Inferred of locations of colibactin alkylation within tested sequence motifs. All alkylated residues are bolded. ICLs and monoadducts are represented by red lines. All data are mean +/- s.d. with n=3 replicates. ****P < 0.0001; not significant (NS), P > 0.05, one-way ANOVA and Tukey's multiple comparison test.

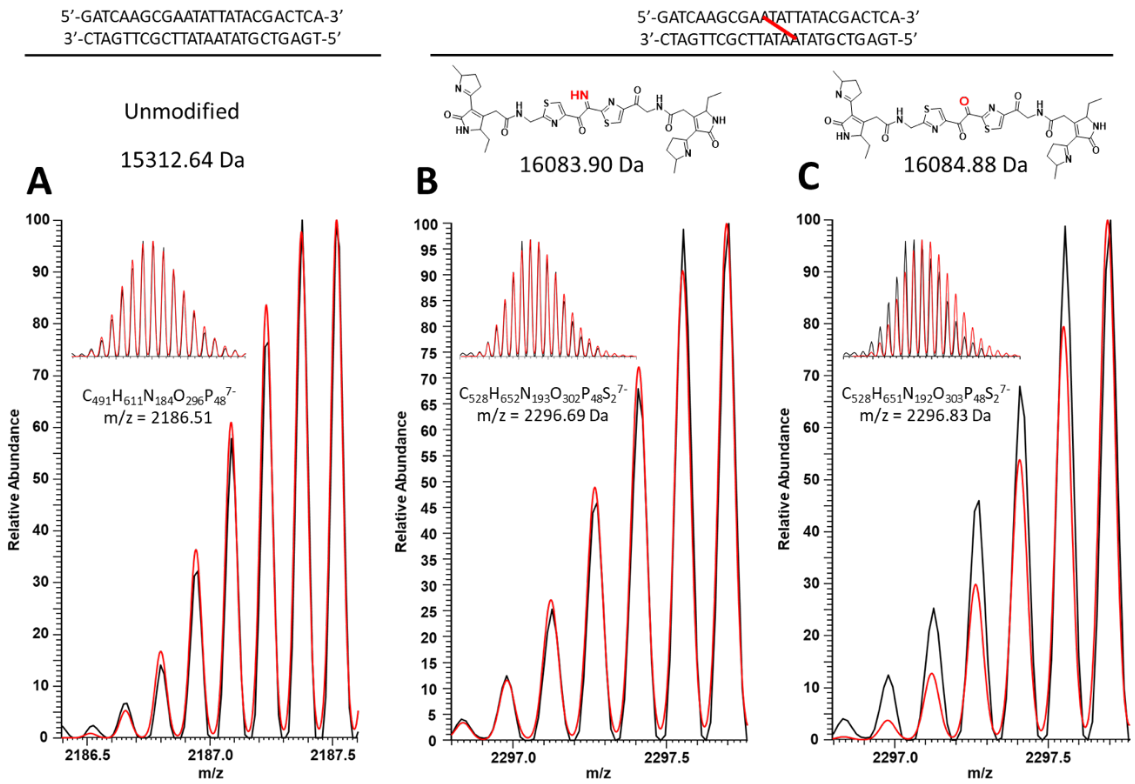

Fig. S12.
HRAM spectra of the 7- charge states of the unmodified 25mer dsODN (A) and colibactin-modified 25mer dsODN (B, C) in black with the simulated spectra in red for the (A) unmodified and modifications of the dsODN with the proposed (B) α-ketoimine and (C) diketone colibactin structures.

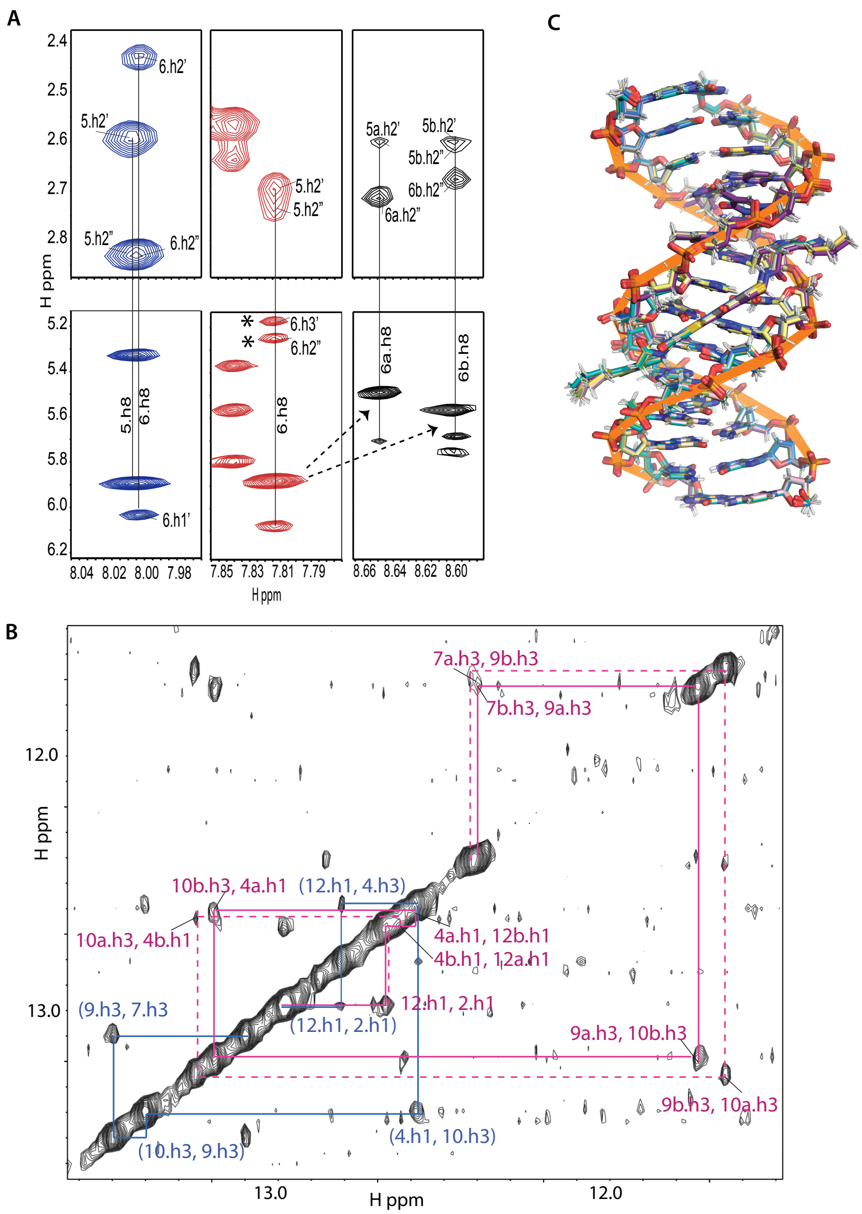

Fig. S13.

NMR analysis of free- and colibactin-bound aDNA. (A) Portion of 2D NOESY experiment of 14-mer DNA (blue), 14-mer DNA with 2'-fluoro-deoxyadenosine at position 6 (red), and the latter crosslinked with colibactin (black). As expected, the 2'-fluoro modification changes the ribose pucker of A6 to 3'-endo, as evidenced by downfield chemical shift of the H2" proton (~5.3 ppm, denoted by asterisk) (*88*). However, upon crosslinking to colibactin, the pucker is shifted back to the typical 2'-endo conformation, as evidenced by the typical chemical shift of H2" of DNA ribose (~2.7 ppm). Furthermore, the change in chemical shift of A6 H8 upon colibactin crosslinking is shown by the dotted arrows. (B) Portion of a 2D NOESY experiment of a mixture of free DNA and colibactin-DNA ICL showing the imino-to-imino walk along the

molecules in blue and magenta, respectively. The chemical shift of T7 and T9 imino protons experience a significant upfield chemical shift upon crosslinking with colibactin. The solid and dashed magenta lines highlight the distinct chemical shifts but similar connectivity patterns obtained due to the pseudosymmetric nature of the colibactin interaction. (**C)** Ensemble of ten NMR restraint derived structures superimposed on DNA atoms in Xplor.

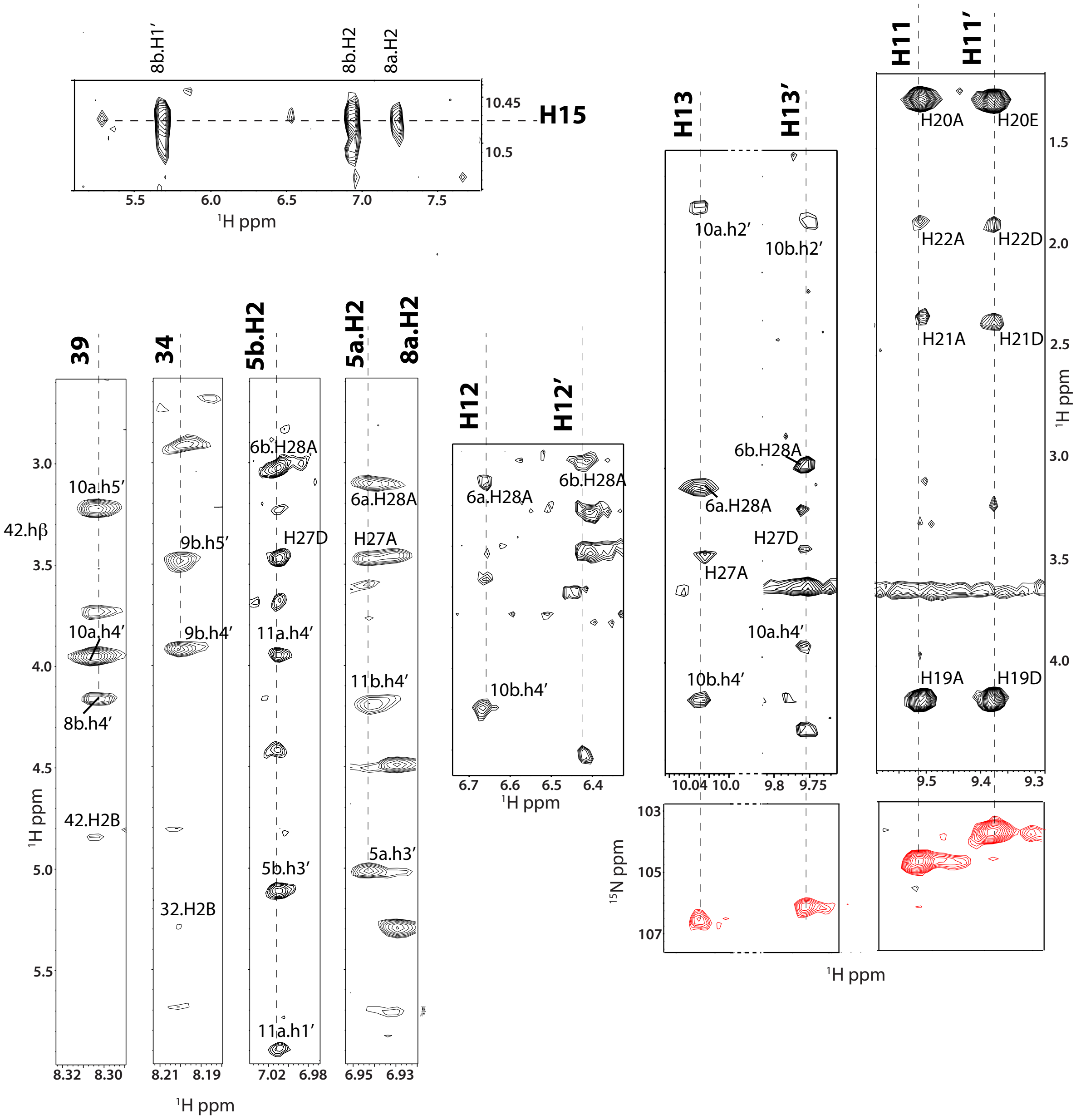

Fig. S14.

Assignment of colibactin proton interactions with the DNA. Portions of a 2D NOESY (black peaks) and 2D HSQC (red peaks) spectra showing intermolecular interaction between the DNA and colibactin. (Top left) N5H protons bound to the central iminium nitrogen show NOE connectivities to the protons of the adenines at the center of the AWWT motif bounding it. (Bottom left) NOE connectivities of thiazole hydrogens C34H and C39H with ribose hydrogens 9b and 10a (H4'/H5'), respectively. (Bottom right) NOE connectivities of NH protons of colibactin.

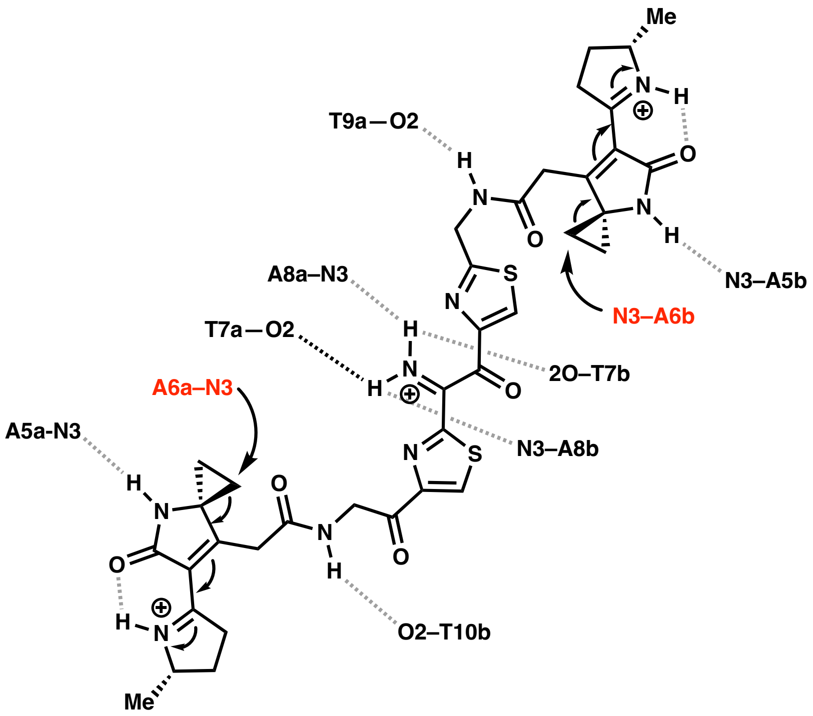

Fig. S15.
Model for DNA interstrand crosslinking by colibactin. Sites of alkylation shown in red.

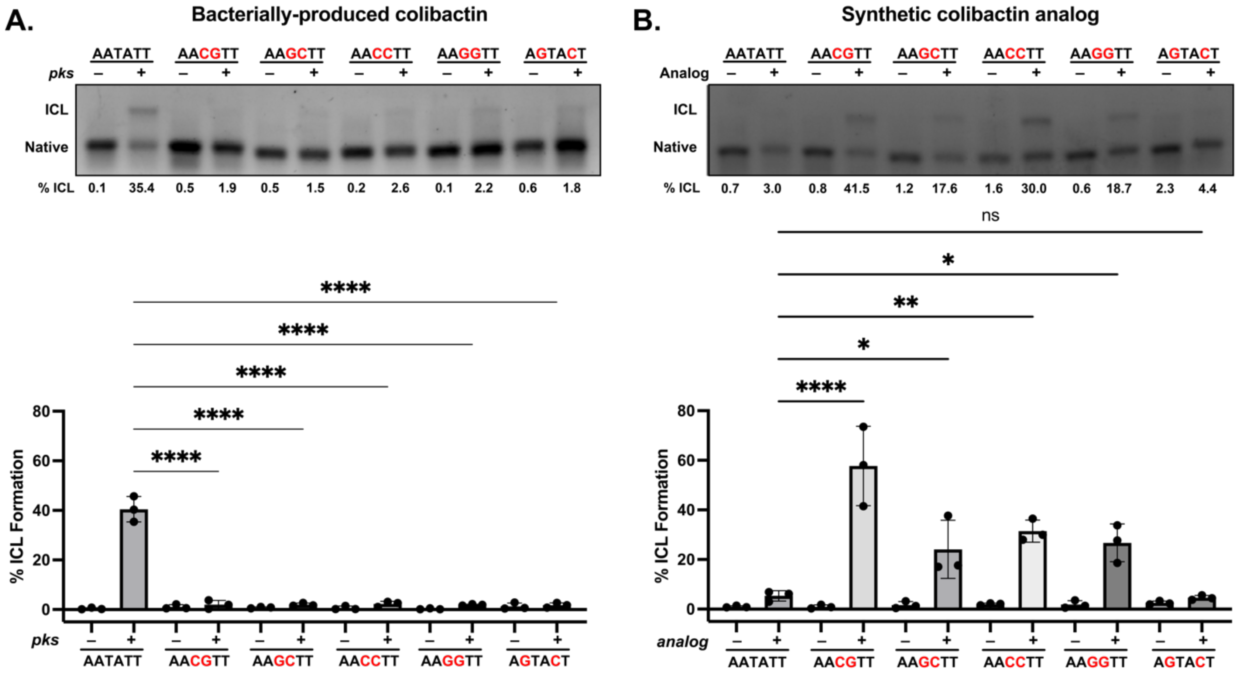

Fig. S16.
The stable colibactin analog preferentially alkylates the AASSTT motif. (A) Quantification of ICL formation by co-incubation with *pks*$^{+}$ *E. coli* using denaturing gel analysis for 50mers containing a permutation of double GC within the sequence 5′-AATATT-3′. Results are quantified through densitometry and shown as bar plots. (C) Quantification of ICL formation by treatment with a colibactin analog using denaturing gel analysis for 50mers containing a permutation of double GC substitution within the sequence 5′-AATATT-3′. Results are quantified through densitometry and shown as bar plots. All data are mean +/- s.d. and n = 3 biological replicates. **** P < 0.0001; ** P < 0.01; * P < 0.05; not significant (ns) P > 0.05, one-way ANOVA and Tukey's multiple comparison test.

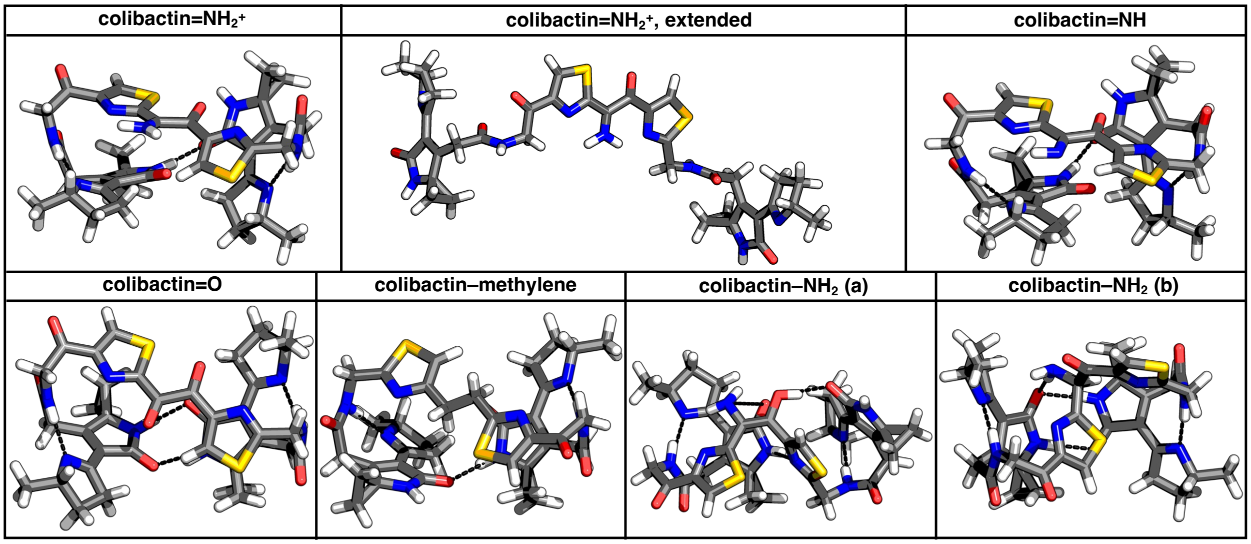

Fig. S17.
Geometries of colibactin structures for which the computed ESP values are shown in main text Fig. 6 and Fig. S18. (Top, left to right) Proposed free colibactin structures with α-ketoiminium (colibactin=NH_2^+), α-ketoiminium in an extended conformation (colibactin=NH_2^+, extended), and α-ketoimine (colibactin=NH) central functional groups, respectively. (Bottom, left to right) Proposed free colibactin structures with diketone (colibactin=O), CH_2–CH_2 (colibactin–methylene), enolamine (colibactin–NH_2, (a)), and aminoketone (colibactin–NH_2, (b)) central functional groups, respectively. Intramolecular hydrogen bonds (HBs) are indicated using black dashed lines. Hydrogen, carbon, nitrogen, oxygen, and sulfur atoms are shown in white, gray, blue, red, and yellow, respectively.

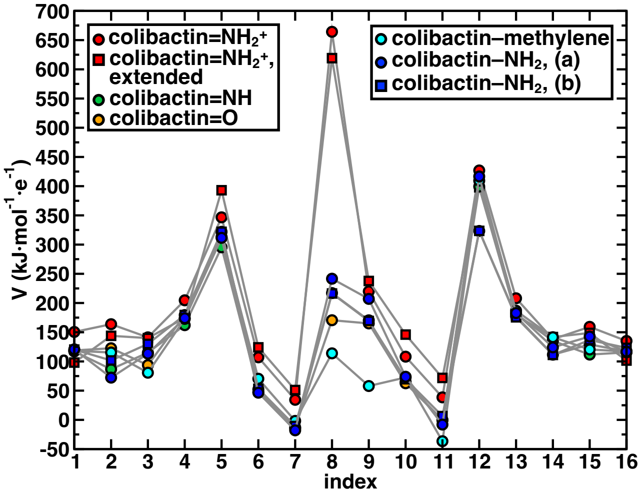

Fig. S18.
Electrostatic potential (ESP) values (V in $kJ \cdot mol^{-1} \cdot e^{-1}$) obtained from density functional theory (DFT) optimization calculations of proposed free colibactin structures at the B3LYP-D3/6-31G* level of theory. ESP values of proposed free colibactin structures with α-ketoiminium central functional group (colibactin=NH_2^+) are shown in red circles (energetically favorable geometry) and red squares (extended geometry observed when colibactin binds to DNA). ESP values of proposed free colibactin structures with α-ketoimine (colibactin=NH), diketone (colibactin=O), CH_2–CH_2 (colibactin–methylene), enolamine (colibactin–NH_2, (a)), and aminoketone (colibactin–NH_2, (b)) central functional groups are shown in green circles, orange circles, cyan circles, blue circles, and blue squares, respectively. The indices 1 through 16 correspond to N1', 27', 28', O (bound to 25'), N2', N4', S (bound to 38 and 39), N37, O (bound to 36), N4, S (bound to 33 and 34), N2, O (bound to 25), 27, 28, and N1 atoms of colibactin, respectively, as labeled in main text Figure 4. The indices 8 and 9 correspond to N37 and O, respectively, for colibactin=NH_2^+, colibactin=NH, colibactin–NH_2 (a), and colibactin–NH_2 (b). The indices 8 and 9 both correspond to O atoms for colibactin=O and C atoms for colibactin–methylene.

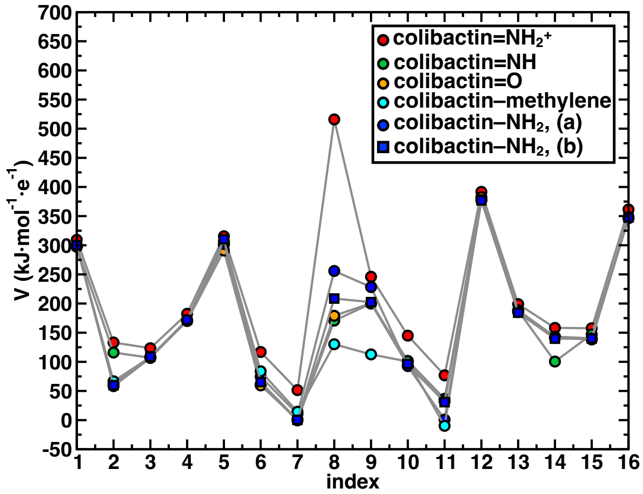

Fig. S19.

Electrostatic potential (ESP) values (V in kJ·mol^{-1}·e^{-1}) obtained from density functional theory (DFT) optimization calculations of proposed colibactin structures crosslinked to the doubly charged DNA sequence "GAATATTC" with 8 base pairs at the B3LYP-D3/6-31G* level of theory. ESP values of proposed colibactin structures with α-ketoiminium (colibactin=NH_2^+), α-ketoimine (colibactin=NH), diketone (colibactin=O), enolamine (colibactin–NH_2, (a)), and aminoketone (colibactin–NH_2, (b)) central functional groups are shown in red circles, green circles, orange circles, cyan circles, blue circles, and blue squares, respectively. The indices 1 through 16 correspond to N1', 27', 28', O (bound to 25'), N2', N4', S (bound to 38 and 39), N37, O (bound to 36), N4, S (bound to 33 and 34), N2, O (bound to 25), 27, 28, and N1 atoms of colibactin, respectively, as labeled in main text Figure 4. The indices 8 and 9 correspond to N37 and O, respectively, for colibactin=NH_2^+, colibactin=NH, colibactin–NH_2 (a), and colibactin–NH_2 (b). The indices 8 and 9 both correspond to O atoms for colibactin=O and C atoms for colibactin–methylene.

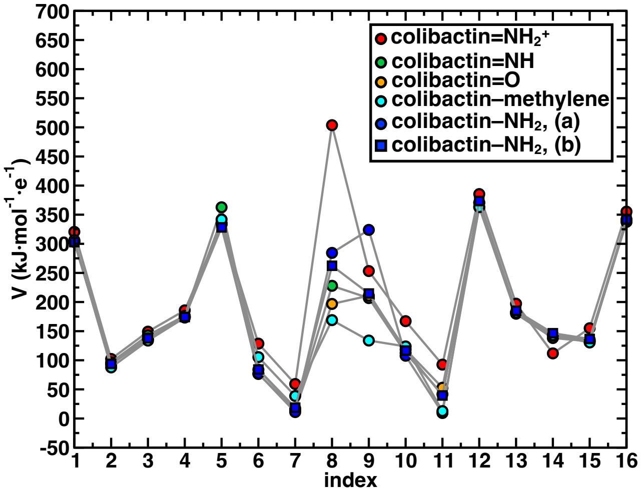

Fig. S20.

Electrostatic potential (ESP) values (V in kJ·mol^{-1}·e^{-1}) obtained from density functional theory (DFT) optimization calculations of proposed colibactin structures crosslinked to the doubly charged DNA mutant sequence “GAACGTTC” with 8 base pairs at the B3LYP-D3/6-31G* level of theory. ESP values of proposed colibactin structures with α-ketoiminium (colibactin=NH_2^+), α-ketoimine (colibactin=NH), diketone (colibactin=O), CH_2–CH_2 (colibactin–methylene), enolamine (colibactin–NH_2, (a)), and aminoketone (colibactin–NH_2, (b)) central functional groups are shown in red circles, green circles, orange circles, cyan circles, blue circles, and blue squares, respectively. The indices 1 through 16 correspond to N1’, 27’, 28’, O (bound to 25’), N2’, N4’, S (bound to 38 and 39), N37, O (bound to 36), N4, S (bound to 33 and 34), N2, O (bound to 25), 27, 28, and N1 atoms of colibactin, respectively, as labeled in main text Figure 4. The indices 8 and 9 correspond to N37 and O, respectively, for colibactin=NH_2^+, colibactin=NH, colibactin–NH_2 (a), and colibactin–NH_2 (b). The indices 8 and 9 both correspond to O atoms for colibactin=O and C atoms for colibactin–methylene.

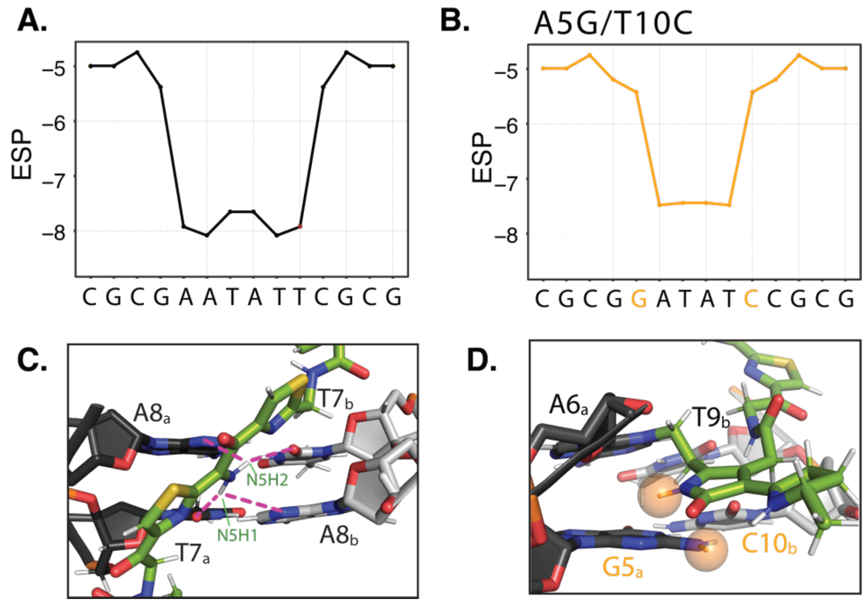

Fig. S21.
Outer base pairs flanking the colibactin alkylation site likely impact colibactin-DNA binding and sequence specificity. (A-B) Electrostatic potential calculations using DNAPhi predicting the highly electronegative environment in the sequence containing the preferred AATATT motif (black) and the decrease in electronegativity upon sequence substitution of the terminal motif base pairs (orange). (C-D) Structural modeling of the equivalent substituted sequences shown in B, showing potential steric clashes between colibactin and DNA (orange spheres).

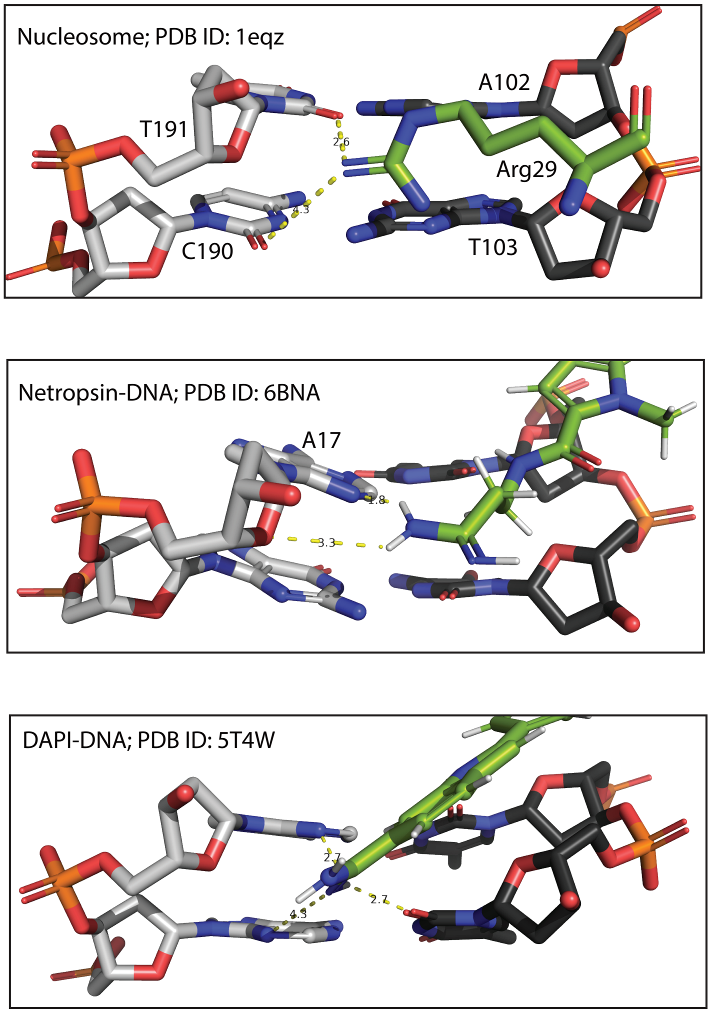

Fig. S22.

Other DNA minor groove binding functional groups resemble interactions of colibactin's central iminium. Top: histone-DNA interaction in the nucleosome shows an arginine moiety deep in the minor groove, with its guanidinium moiety in between AT sequence. The arginine guanidinium moiety also has a high pKa (~13.8) (*16*) rendering it protonated at physiological pH, in which case, one of the protons in this example would form a hydrogen bond, whereas the other would make electrostatic interaction. Middle: structure of netropsin complexed with DNA shows the amino group of the terminal amidinium making hydrogen bond and electrostatic interaction with N3 aromatic ring and O of ribose ring, respectively. Bottom: structure of DAPI complexed with DNA shows the terminal amino group in between an AA sequence making electrostatic interaction with N3 of adenine and O2 of thymine of the opposing strand. These examples highlight the similarity between the α-ketoiminium nitrogen atom in colibactin and the protonated nitrogen atoms in DNA-binding protein side chains and small molecules that allow for interaction with electronegative, AT-rich minor grooves in DNA.

Table S1.
Statistics for the colibactin-DNA ICL structure.

	**DNA-COLIBACTIN**
**NMR distance and dihedral constraints**	
**Distance Restraints**	
**Total NOE**	355
Intraresidue	104
Sequential ($\|i-j\|=1$)	103
Inter-chain	64
Hydrogen bonds	84
**Total dihedral-angle restraints**	9
**Structure statistics (ensemble of 10)**	
Violations (> 2 Å)	60 (1.7%)
Distance constraints (Å)	0.3 ± 0.1
Max. distance-constraint violation (Å)	0.56
Dihedral-angle constraints (°)	2.87 ± 1.5
Max. dihedral-angle violation (°)0	6.7
**Deviations from idealized geometry**	
Bond lengths (Å)	0
Bond angles (°)	43.9 ± 2.5 (2.3%)
Impropers (°)	9.9 ± 2.6 (1.4%)
**Average pairwise r.m.s. deviation (Å)***	
DNA-Colibactin	0.55 ± 0.11

Table S2.

Chemical shifts of colibactin atoms (ppm)

Hydrogen atoms are labeled with the number of the atom to which they are connected (see Fig. 4 for numbering scheme). A,B,C to designate assignment to an atom from the northern warhead (plain numbering) and D,E,F to designate assignment to an atom from the southern warhead (' numbering).

C19	27.6783	H20E	1.3508
C19'	27.6989	H20F	1.3508
C20	22.6735	H21A	2.3916
C20'	22.651	H21B	2.2222
C21	35.5276	H21D	2.4226
C21'	35.5127	H21E	2.1361
C22	29.6896	H22A	1.9295
C22'	29.7442	H22B	1.9645
C30	42.1679	H22D	1.946
C30'	42.0168	H22E	1.8845
C32	34.6591	H27A	3.5408
C34	126.1428	H27B	3.5408
C39	128.9894	H27D	3.5223
C42	44.9621	H27E	3.5223
C19	27.6783	H30A	3.1873
C19'	27.6989	H30B	3.4086
H11	9.5051	H30D	3.3318
H11'	9.3768	H30E	3.3142
H12	6.7733	H32A	5.3239
H12'	6.5133	H32B	5.3161
H13	10.0301	H34	8.193
H13'	9.7575	H39	8.2988
H15	10.4693	H42A	2.893
H16	10.5035	H42B	4.8562
H19A	4.2381	N1	104.7411
H19D	4.2281	N1'	103.7926
H20A	1.3487	N2	118.726
H20B	1.3487	N2'	113.0451
H20C	1.3487	N3	106.5892
H20D	1.3508	N3'	106.1613
		N37	82.0684

Table S3.
Annealed oligonucleotides used in DNA crosslinking experiments.

Name	Sequence
AAATTAATA (50 bp)	5-GATCTCGATCCCGCGAAATTAATACGACTCACTATAGGGGAATTGTGAGC-3<br>3-CTAGAGCTAGGGCGCTTTAATTATGCTGAGTGATATCCCCTTAACACTCG-5
AATATTATA (50 bp)	5-GATCTCGATCCCGCGAATATTATACGACTCACTATAGGGGAATTGTGAGC-3<br>3-CTAGAGCTAGGGCGCTTATAATATGCTGAGTGATATCCCCTTAACACTCG-5
ATTAATATA (50 bp)	5-GATCTCGATCCCGCGATTAATATACGACTCACTATAGGGGAATTGTGAGC-3<br>3-CTAGAGCTAGGGCGCTAATTATATGCTGAGTGATATCCCCTTAACACTCG-5
ATTAATATA (50 bp)	5-GATCTCGATCCCGCGATTAATAAACGACTCACTATAGGGGAATTGTGAGC-3<br>3-CTAGAGCTAGGGCGCTAATTATTTGCTGAGTGATATCCCCTTAACACTCG-5
FWD-FAM-AAATTAATA (50 bp)	FAM-5-GATCTCGATCCCGCGAAATTAATACGACTCACTATAGGGGAATTGTGAGC-3<br>3-CTAGAGCTAGGGCGCTTTAATTATGCTGAGTGATATCCCCTTAACACTCG-5
FWD-FAM-AATATTATA (50 bp; FAM: 6-carboxyfluorescein)	FAM-5-GATCTCGATCCCGCGAATATTATACGACTCACTATAGGGGAATTGTGAGC-3<br>3-CTAGAGCTAGGGCGCTTATAATATGCTGAGTGATATCCCCTTAACACTCG-5
FWD-FAM-ATTAATATA (50 bp; FAM: 6-carboxyfluorescein)	FAM-5-GATCTCGATCCCGCGATTAATATACGACTCACTATAGGGGAATTGTGAGC-3<br>3-CTAGAGCTAGGGCGCTAATTATATGCTGAGTGATATCCCCTTAACACTCG-5
FWD-FAM-ATTAATATA (50 bp; FAM: 6-carboxyfluorescein)	FAM-5-GATCTCGATCCCGCGATTAATAAACGACTCACTATAGGGGAATTGTGAGC-3<br>3-CTAGAGCTAGGGCGCTAATTATTTGCTGAGTGATATCCCCTTAACACTCG-5
REV-FAM-AAATTAATA (50 bp; FAM: 6-carboxyfluorescein)	5-GATCTCGATCCCGCGAAATTAATACGACTCACTATAGGGGAATTGTGAGC-3<br>3-CTAGAGCTAGGGCGCTTTAATTATGCTGAGTGATATCCCCTTAACACTCG-5-FAM
REV-FAM-AATATTATA (50 bp; FAM: 6-carboxyfluorescein)	5-GATCTCGATCCCGCGAATATTATACGACTCACTATAGGGGAATTGTGAGC-3<br>3-CTAGAGCTAGGGCGCTTATAATATGCTGAGTGATATCCCCTTAACACTCG-5-FAM
REV-FAM-ATTAATATA (50 bp; FAM: 6-carboxyfluorescein)	5-GATCTCGATCCCGCGATTAATATACGACTCACTATAGGGGAATTGTGAGC-3<br>3-CTAGAGCTAGGGCGCTAATTATATGCTGAGTGATATCCCCTTAACACTCG-5-FAM
REV-FAM-ATTAATATA (50 bp; FAM: 6-carboxyfluorescein)	5-GATCTCGATCCCGCGATTAATAAACGACTCACTATAGGGGAATTGTGAGC-3<br>3-CTAGAGCTAGGGCGCTAATTATTTGCTGAGTGATATCCCCTTAACACTCG-5-FAM
AAATTAATA (25 bp)	5-GATCAAGCGAAATTAATACGACTCA-3<br>3-CTAGTTCGCTTTAATTATGCTGAGT-5
AATATTATA (25 bp)	5-GATCAAGCGAATATTATACGACTCA-3<br>3-CTAGTTCGCTTATAATATGCTGAGT-5
ATTAATATA (25 bp)	5-GATCAAGCGATTAATATACGACTCA-3<br>3-CTAGTTCGCTAATTATATGCTGAGT-5
ATTAATAAA (25 bp)	5-GATCAAGCGATTAATAAACGACTCA-3<br>3-CTAGTTCGCTAATTATTTGCTGAGT-5
GATATT (50 bp)	5-GATCTCGATCCCGCGGATATTATACGACTCACTATAGGGGAATTGTGAGC-3<br>3-CTAGAGCTAGGGCGCCTATAATATGCTGAGTGATATCCCCTTAACACTCG-5
AGTATT (50 bp)	5-GATCTCGATCCCGCGAGTATTATACGACTCACTATAGGGGAATTGTGAGC-3<br>3-CTAGAGCTAGGGCGCTCATAATATGCTGAGTGATATCCCCTTAACACTCG-5
AACATT (50 bp)	5-GATCTCGATCCCGCGAACATTATACGACTCACTATAGGGGAATTGTGAGC-3<br>3-CTAGAGCTAGGGCGCTTGTAATATGCTGAGTGATATCCCCTTAACACTCG-5
AATGTT (50 bp)	5-GATCTCGATCCCGCGAATGTTATACGACTCACTATAGGGGAATTGTGAGC-3<br>3-CTAGAGCTAGGGCGCTTACAATATGCTGAGTGATATCCCCTTAACACTCG-5
AATACT (50 bp)	5-GATCTCGATCCCGCGAATACTATACGACTCACTATAGGGGAATTGTGAGC-3<br>3-CTAGAGCTAGGGCGCTTATGATATGCTGAGTGATATCCCCTTAACACTCG-5
AATATC (50 bp)	5-GATCTCGATCCCGCGAATATCATACGACTCACTATAGGGGAATTGTGAGC-3<br>3-CTAGAGCTAGGGCGCTTATAGTATGCTGAGTGATATCCCCTTAACACTCG-5
GATATT (25 bp)	5-GATCAAGCGGATATTATACGACTCA-3<br>3-CTAGTTCGCCTATAATATGCTGAGT-5
AGTATT (25 bp)	5-GATCAAGCGAGTATTATACGACTCA-3<br>3-CTAGTTCGCTCATAATATGCTGAGT-5
AACATT (25 bp)	5-GATCAAGCGAACATTATACGACTCA-3

	3-CTAGTTCGCTTGTAATATGCTGAGT-5
AATGTT (25 bp)	5-GATCAAGCGAATGTTATACGACTCA-3<br>3-CTAGTTCGCTTACAATATGCTGAGT-5
AATACT (25 bp)	5-GATCAAGCGAATACTATACGACTCA-3<br>3-CTAGTTCGCTTATGATATGCTGAGT-5
AATATC (25 bp)	5-GATCAAGCGAATATCATACGACTCA-3<br>3-CTAGTTCGCTTATAGTATGCTGAGT-5
FWD-deaza (50 bp;<br>**X** = *N*3-deaza-dAdo)	5-GATCTCGATCCCGCGA**X**TATTATACGACTCACTATAGGGGAATTGTGAGC-3<br>3-CTAGAGCTAGGGCGCTTATAATATGCTGAGTGATATCCCCTTAACACTCG-5
REV-deaza (50 bp;<br>**X** = *N*3- deaza-dAdo)	5-GATCTCGATCCCGCGAATATTATACGACTCACTATAGGGGAATTGTGAGC-3<br>3-CTAGAGCTAGGGCGCTTAT**X**ATATGCTGAGTGATATCCCCTTAACACTCG-5
FWD/REV-deaza (50 bp;<br>**X** = *N*3-deaza-dAdo	5-GATCTCGATCCCGCGA**X**TATTATACGACTCACTATAGGGGAATTGTGAGC-3<br>3-CTAGAGCTAGGGCGCTTAT**X**ATATGCTGAGTGATATCCCCTTAACACTCG-5
2′-fluoro-14mer used for NMR (**X** = 2′-fluoro-dAdo)	5-CGCGA**X**TATTCGCG-3<br>3-GCGCTTAT**X**AGCGC-5
Unmodified 14mer used for NMR	5-CGCGAATATTCGCG-3<br>3-GCGCTTATAAGCGC-5
GACATT (50 bp)	5-GATCTCGATCCCGCGGACATTATACGACTCACTATAGGGGAATTGTGAGC-3<br>3-CTAGAGCTAGGGCGCCTGTAATATGCTGAGTGATATCCCCTTAACACTCG-5
GATCTT (50 bp)	5-GATCTCGATCCCGCGGATCTTATACGACTCACTATAGGGGAATTGTGAGC-3<br>3-CTAGAGCTAGGGCGCCTAGAATATGCTGAGTGATATCCCCTTAACACTCG-5
GATATC (50 bp)	5-GATCTCGATCCCGCGGATATCATACGACTCACTATAGGGGAATTGTGAGC-3<br>3-CTAGAGCTAGGGCGCCTACAGTATGCTGAGTGATATCCCCTTAACACTCG-5
AGTACT (50 bp)	5-GATCTCGATCCCGCGAGTACTATACGACTCACTATAGGGGAATTGTGAGC-3<br>3-CTAGAGCTAGGGCGCTCACGATATGCTGAGTGATATCCCCTTAACACTCG-5
AACGTT (50 bp)	5-GATCTCGATCCCGCGAACGTTATACGACTCACTATAGGGGAATTGTGAGC-3<br>3-CTAGAGCTAGGGCGCATGCAATATGCTGAGTGATATCCCCTTAACACTCG-5
AAGCTT (50 bp)	5-GATCTCGATCCCGCGAAGCTTATACGACTCACTATAGGGGAATTGTGAGC-3<br>3-CTAGAGCTAGGGCGCATCGAATATGCTGAGTGATATCCCCTTAACACTCG-5
AACCTT (50 bp)	5-GATCTCGATCCCGCGAACCTTATACGACTCACTATAGGGGAATTGTGAGC-3<br>3-CTAGAGCTAGGGCGCATGGAATATGCTGAGTGATATCCCCTTAACACTCG-5
AAGGTT (50 bp)	5-GATCTCGATCCCGCGAAGGTTATACGACTCACTATAGGGGAATTGTGAGC-3<br>3-CTAGAGCTAGGGCGCATCCAATATGCTGAGTGATATCCCCTTAACACTCG-5

Table S4. Electrostatic potential (ESP) values (in $kJ \cdot mol^{-1} \cdot e^{-1}$) computed for specific atoms on doubly charged DNA sequence "GAATATTC" with 8 base pairs which is crosslinked by colibactin with α-ketoiminium (colibactin=NH_2^+), α-ketoimine (colibactin=NH), diketone (colibactin=O), CH_2–CH_2 (colibactin–methylene), enolamine (colibactin–NH_2, enolamine), and aminoketone (colibactin–NH_2, aminoketone) central functional groups.

index	colibactin=NH_2^+	colibactin=NH	colibactin=O	colibactin–methylene	colibactin–NH_2, enolamine	colibactin–NH_2, aminoketone
THY 107 O2	281.46	251.04	252.25	255.08	258.12	257.06
ADE 108 N3	207.42	174.16	167.98	168.58	175.12	177.72
ADE 108 O4' (ribose)	202.20	169.15	167.78	168.80	169.03	169.07
THY 109 O2	288.31	267.63	263.69	262.70	260.56	262.81
THY 110 O2	284.46	269.87	269.18	269.86	270.64	271.63
THY 207 O2	284.45	264.03	260.55	261.40	263.20	264.50
ADE 208 N3	199.31	169.87	167.27	177.07	175.51	176.76
ADE 208 O4' (ribose)	206.49	185.31	180.87	180.55	181.46	180.58
THY 209 O2	281.77	257.86	259.20	259.90	258.69	258.54
THY 210 O2	284.92	268.66	268.62	269.48	269.79	268.80

Table S5. Hydrogen bond (HB) interaction energies (E_{int} in kcal/mol) corresponding to inter-molecular HBs between colibactin and DNA, and intramolecular HBs of colibactin crosslinked to doubly charged DNA sequence "GAATATTC" with 8 base pairs. These energies are obtained for alkylated colibactin with α-ketoiminium (colibactin=NH_2^+), α-ketoimine (colibactin=NH), diketone (colibactin=O), CH_2–CH_2 (colibactin–methylene), and enolamine (colibactin–NH_2, enolamine) central functional groups. HB interaction energies of HBs involving the central functional groups are indicated in column 2, and the total HB interaction energy of all inter- and intra-molecular HBs is indicated in column 3. The HB interaction energy was quantified using Multiwfn (*78*) through the presence of bond critical points (BCPs) (*89*) from the quantum theory of atoms in molecules (QTAIM) (*90*) and the corresponding potential energy density (*91*).

**DNA-colibactin complex**	**E_{int} involving central functional groups (kcal/mol)**	**Total E_{int} (kcal/mol)**
colibactin=NH_2^+	-14.26	-33.78
colibactin=NH	-9.66	-33.58
colibactin=O	0.00	-13.78
colibactin–methylene	0.00	-10.29
colibactin–NH_2, enolamine	-7.27	-20.00

Data S1.
Structural coordinates of DFT-optimized structures of colibactin.
